# A permutation method for network assembly

**DOI:** 10.1371/journal.pone.0240888

**Published:** 2020-10-23

**Authors:** Shawn A. Means, Christian Bläsche, Carlo R. Laing

**Affiliations:** School of Natural and Computational Sciences, Massey University, Auckland, New Zealand; University of Sussex, UNITED KINGDOM

## Abstract

We present a method for assembling directed networks given a prescribed bi-degree (in- and out-degree) sequence. This method utilises permutations of initial adjacency matrix assemblies that conform to the prescribed in-degree sequence, yet violate the given out-degree sequence. It combines directed edge-swapping and constrained Monte-Carlo edge-mixing for improving approximations to the given out-degree sequence until it is exactly matched. Our method permits inclusion or exclusion of ‘multi-edges’, allowing assembly of weighted or binary networks. It further allows prescribing the overall percentage of such multiple connections—permitting exploration of a weighted synthetic network space unlike any other method currently available for comparison of real-world networks with controlled multi-edge proportion null spaces. The graph space is sampled by the method non-uniformly, yet the algorithm provides weightings for the sample space across all possible realisations allowing computation of statistical averages of network metrics as if they were sampled uniformly. Given a sequence of in- and out- degrees, the method can also produce simple graphs for sequences that satisfy conditions of graphicality. Our method successfully builds networks with order *O*(10^7^) edges on the scale of minutes with a laptop running Matlab. We provide our implementation of the method on the GitHub repository for immediate use by the research community, and demonstrate its application to three real-world networks for null-space comparisons as well as the study of dynamics of neuronal networks.

## Introduction

Interactions between entities as disparate as genes, computers, infected people, predators and prey or neurons of the brain are readily represented with networks [1–6]. Comprised of nodes and edges connecting them, networks—or graphs—are naturally of great interest for the study of such myriad systems, and, given enough detail of the underlying structure, provide an essential framework for analysing the complex dynamics emerging on the network topology [7]. Therein lies a challenge, however: details of the network structure for a given system are often limited to only the number of connections between entities, otherwise known as the node-degrees. These may represent the number of sexual partners [4], number of prey species tangled in an ecological food web [5], or number of outbound and inbound synaptic connections between neurons [8]. Generating an actual network with a given sequence of node-degrees itself can pose a challenging task, particularly if the resulting network is forbidden from including multiple connections between nodes or loop-backs from a node to itself; such a constrained network is otherwise known as a ‘simple graph’. Additionally, a single degree sequence may realise numerous networks; inspecting the influence of network topology on a system knowing only the degree sequence often compels appropriate sampling for the space of possible network realisations to avoid introduction of bias. Moreover, sampling the space allowing inspection of statistical properties for real-world networks requires comparison to baseline ‘null’ models. These samples permit determination of statistical significance for real-world network characteristics such as assortativity or tendency of nodes to establish links with similar nodes; naturally, this entails generating suites of synthetic networks for comparison.

These network generation challenges have attracted considerable attention. Well-known methods such as the Configuration Model (CM) [9] combined with Monte-Carlo Markov Chain random-swapping for sampling the graph space that match given degree sequences are utilised, but can be computationally expensive. Rapid alternatives such as the Chung-Lu method satisfy the expected distribution of degree sequences, and can be quite suitable if matching the sequences exactly is not required [10]. Variants with improvements are steadily presented and analysed in the literature such as the extension of Chung-Lu to include multi-edges via hypergeometric distributions [11], or the ‘soft’ CM aiming at meeting degree distributions instead of the exact sequences [12] (see [13] for a recent overview). However, we found no assembly method capable of not only exactly fulfilling a given degree sequence, but also that provides any control over the resulting proportion of multiple edges—beyond their exclusion altogether. Attention to the reciprocity of networks, or directed edges establishing connections both ways between nodes [14] and the multiplicities of edges, or formation of triangles between vertices [15], are related but do not address this issue. We thus devised a novel scheme for assembling networks that we call the ‘permutation’ method—since it relies on permuting entries of an initial connectivity matrix, or the so-called adjacency matrix, representing the connections between nodes. We present this method with application to ‘real-world’ network degree sequences for generation of null-model comparisons, and further present an application to our study of dynamics on neuronal networks [7].

Note, we did not craft this method to explicitly address uniform sampling of the possible realisation space, nor the substantial theoretical work already performed in this arena [16–20]. We do parsimoniously exploit some theorems for ensuring graphicality, or simply that a given degree sequence may realise a bona fide network [21]. Nevertheless, some analysis for simple small networks (*N* < 10) and performance with larger networks of interest for our neuronal studies (*N* ∈ *O*(10^4^)) is presented. We further provide a function written in Matlab (GitHub repository [22]) demonstrating this permutation method that accepts degree sequences, the desired proportion of multiple-edge connections, and optionally returns estimates for the weighted non-uniform sampling of the given graph space.

## Materials and methods

We consider directional networks, or ‘bigraphs’, designated by two degree sequences: an *in* degree (i.e., synaptic inputs) and an *out* degree (synaptic outputs) which we denote **k**_**in**_ and **k**_**out**_, respectively. We can for instance designate desired **k**_**in**_ and **k**_**out**_ sequences (whose sums are equal—see below) for *N* nodes in a network with perhaps a desired correlation between an individual node’s in- and out-degrees, or generated according to, say, a power-law distribution. And, once these sequences are so designated, the task of connecting the nodes or wiring up the network remains. We thus here present a method for assembling a network given these desired, or ‘target’ **k**_**in**_ and **k**_**out**_ sequences that optionally excludes multi-edges and self-loops if desired, or, alternatively, attempts to meet a target proportion of multiple connections. Assuming said sequences of node in- and out-degrees are already provided in **k**_**in**_ and **k**_**out**_, we omit discussion of generating correlated sequences or other properties of interest (see for instance [7] or [23]) and instead focus on utilising already prescribed bi-graph sequences in the network assembly.

**Fig 1:**
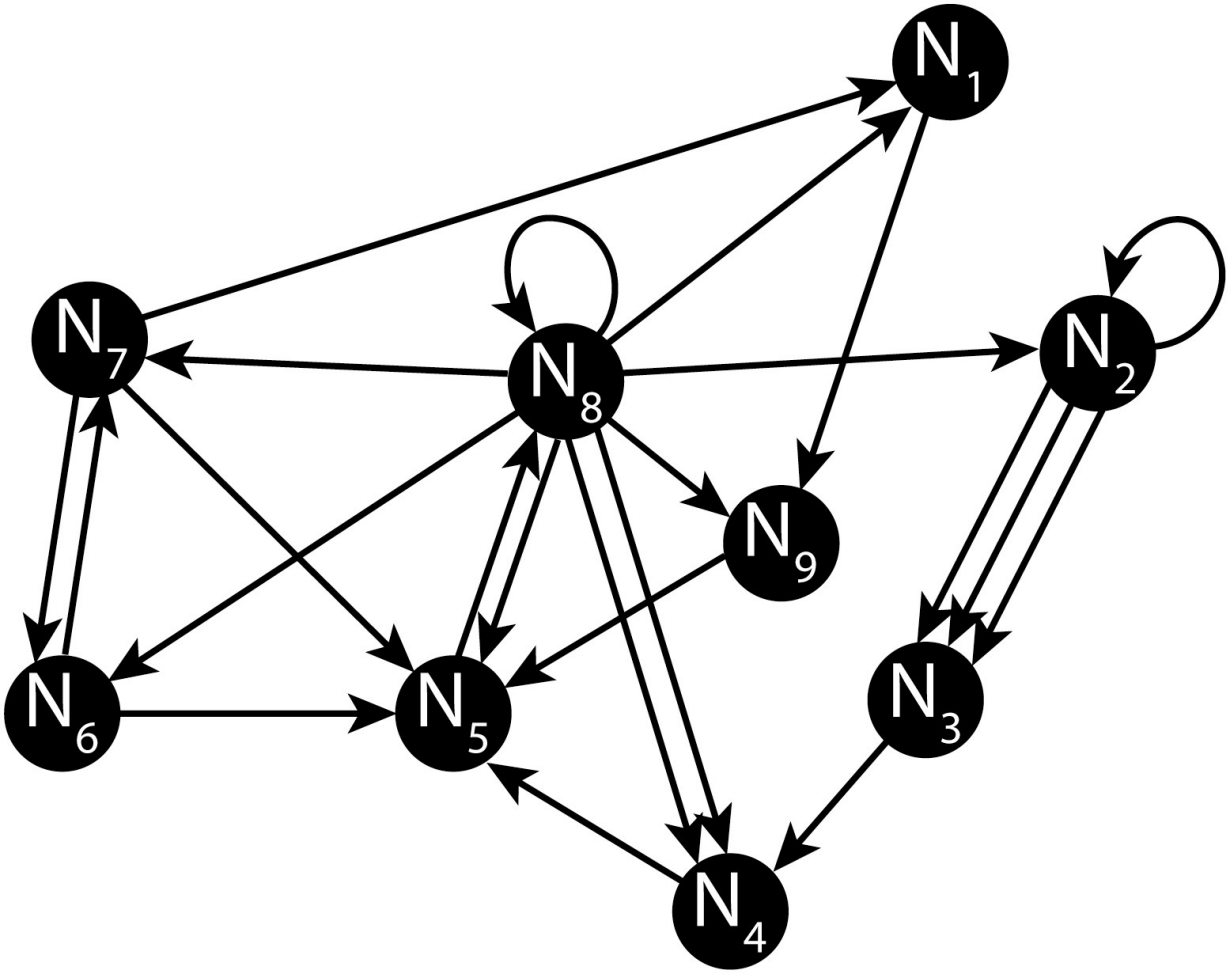
Example model network. An example network with *N* = 9 nodes and directional edges connecting them in a ‘Directed Graph’ with directionality corresponding to flow of neuronal excitation. Note some edges are self-loops (e.g., Node 2 and 8), and multiple-connects exist between others (e.g., Node 2 and Node 3). This is therefore *not* a ‘simple’ graph since it includes auto- and multiple-connections.

The example network schematic shown in Fig 1 corresponds to the in and out degree sequences:

$$\begin{array}{cc} k_{\text{in}} & =[\begin{array}{ccccccccc} 2 & 2 & 3 & 3 & 5 & 2 & 2 & 2 & 2 \end{array}] \end{array}$$ (1)

$$\begin{array}{cc} k_{\text{out}} & =[\begin{array}{ccccccccc} 1 & 4 & 1 & 1 & 1 & 2 & 3 & 9 & 1 \end{array}]. \end{array}$$ (2)

The particular realisation for the network of Fig 1 is conveniently captured in the adjacency matrix, **A**, whose row entries correspond to the inbound edges and column entries correspond to the outbound as follows:

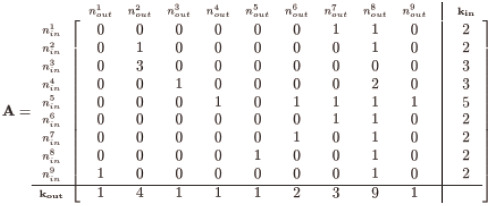
(3)
and note the appended column and row for the corresponding **k**_**in**_ and **k**_**out**_ is simply the row or column sums across **A**, respectively. Hence, node #8 in our demonstration network of Fig 1 has in-degree of 2 and out-degree of 9. Self-loops in the network are further easily identified by inspecting the diagonal of **A** for nonzero entries, appearing here for nodes 2 and 8. Note, this is only one of many possible realisations for the given sequences of **k**_**in**_ and **k**_**out**_; in general, there are multitudes of adjacency matrices corresponding to given degree sequences, and this particular **A** is merely one instance.

### The permutation method

#### Initialisation

With the demonstration **k**_**in**_ and **k**_**out**_ as given in Eq (3), it is a straightforward affair to assemble a precursor adjacency matrix, which we denote **A**^(0)^, with entries consisting only of ‘0’s and ‘1’s thus:

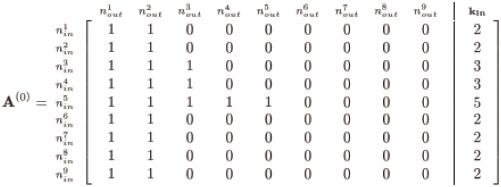
(4)

Notice we insert a number of solo connections (represented as ‘1’s) corresponding to the in-degree for each node—for instance the 5 ones for node 5’s *k*_*in*_ (labeled as $n_{in}^{5}$)—and pad the remaining row vector entries with ‘0’s representing the lack of an edge. Although this satisfies each node’s designated *k*_*in*_, we do not concern ourselves with satisfying *k*_*out*_—yet. Our initial **A**^(0)^ is then permuted row-by-row to randomly position the edges represented by the ‘1’s such that all the nodes’ *k*_*in*_ degrees are still satisfied but with a now randomly distributed structure along the rows:

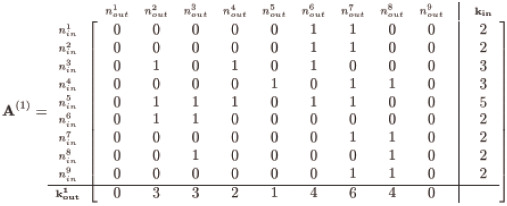
(5)

The above instance (generated via Matlab’s randperm) is but one possible form; nevertheless, the method maintains adherence to the given **k**_**in**_ for any random permutation since we permute entries by row. We now have two adjacency matrices: an **A**^(0)^ with a trivial assembly, and an **A**^(1)^ permutation of row entries of **A**^(0)^, both of which satisfy the given **k**_**in**_ yet violate the designated **k**_**out**_—unless we are spectacularly fortunate and the row permutations match **k**_**out**_.

#### The algorithm

Disregarding the designated **k**_**out**_, however, gives us an ‘actual’ $k_{\text{out}}^{1}$ that in all likelihood is quite incorrect: compare with the given **k**_**out**_ in Eq (3). At this point, the method now begins its work by manipulating entries of **A**^(1)^ to reduce the error of out-degrees until we hit the given or target **k**_**out**_ all the while adhering to **k**_**in**_. We calculate an out-degree error, $k_{\text{out}}^{r}$, for an *i*^*th*^ iteration of a sequence of interim adjacency matrices we denote **A**^(*i*)^, by comparing the current $k_{\text{out}}^{i}$ with the target:

$$\begin{array}{cc} k_{\text{out}}^{r} & =k_{\text{out}}^{i}-k_{\text{out}}^{t}\\ r_{out} & =\left|\right|k_{\text{out}}^{r}{\left|\right|}_{2}, \end{array}$$ (6)

where $k_{\text{out}}^{t}$ is the target out-degree. Computing the *L*^2^-norm of the difference gives us a metric for ‘convergence’: i.e., if *r*_*out*_ < *ϵ*, we consider the procedure successful for some error tolerance, *ϵ*.

**Fig 2:**
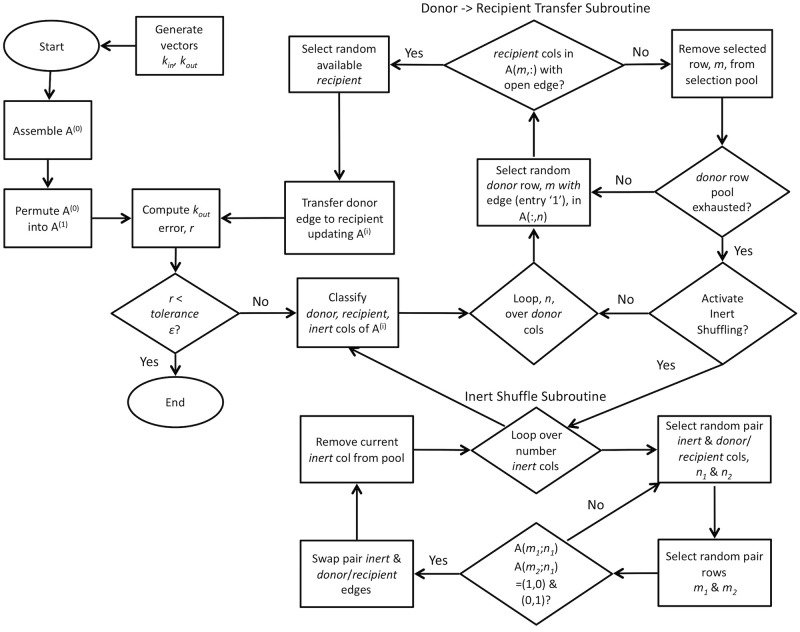
Flowchart schematic illustrating method for manipulating entries of A^(1)^ to satisfy both k_in_ and k_out_. Once provided a sequence of node degrees in *k*_*in*_ and *k*_*out*_, the method classifies which nodes are above (*donor*), below (*recipient*) and at (*inert*) the target $k_{\text{out}}^{t}$, and proceeds to transfer surplus edges from *donors* to *recipients*. If enough failures to match suitable edges from the pool of *donors* to *recipients* occur, shuffling of edges from the set of *inert* nodes is performed. The process continues until deviation from $k_{\text{out}}^{t}$ falls below acceptable tolerance, *ϵ*, and we obtain a final adjacency matrix **A**^(**f**)^.

The procedure for manipulating **A**^(*i*)^ is key, and is illustrated in the somewhat daunting flowchart of Fig 2; we clarify some of the terminology here. The method hinges on the feature of **A**^(**1**)^ such that each nodal $k_{out}^{i}$ is either above, below or precisely at the target value given in $k_{\text{out}}^{t}$. We classify these as ‘*donor*’, ‘*recipient*’ and ‘*inert*’ nodes, respectively, and illustrate this aspect by computing the difference between $k_{\text{out}}^{1}$ in Eq (5) and $k_{\text{out}}^{t}$ for our example network:

$$\begin{array}{cc} k_{\text{out}}^{r} & =k_{\text{out}}^{1}-k_{\text{out}}^{t}\\ k_{\text{out}}^{r} & =[\begin{array}{ccccccccc} 0 & 3 & 3 & 2 & 1 & 4 & 6 & 4 & 0 \end{array}]-[\begin{array}{ccccccccc} 1 & 4 & 1 & 1 & 1 & 2 & 3 & 9 & 1 \end{array}]\\ & =[\begin{array}{ccccccccc} -1 & -1 & 2 & 1 & 0 & 2 & 3 & -5 & -1 \end{array}]. \end{array}$$ (7)

Each entry in $k_{\text{out}}^{r}$ indicates the current out-degree deviation as having too many connections (e.g., node #3 with two extra outbound edges), too few connections (e.g., node #8 with five unfilled edge stubs) or just enough (e.g., node #5). The essence of the method is simply this: transfer surplus outbound edges from *donor* nodes with too many edges to *recipient* nodes with too few. This transfer from the pool of *donor* edges to *recipients* shifts $k_{\text{out}}^{i}$ closer to the target, all the while constraining selection of suitable transfers such that no alteration of **k**_**in**_ is permitted.

**Fig 3:**
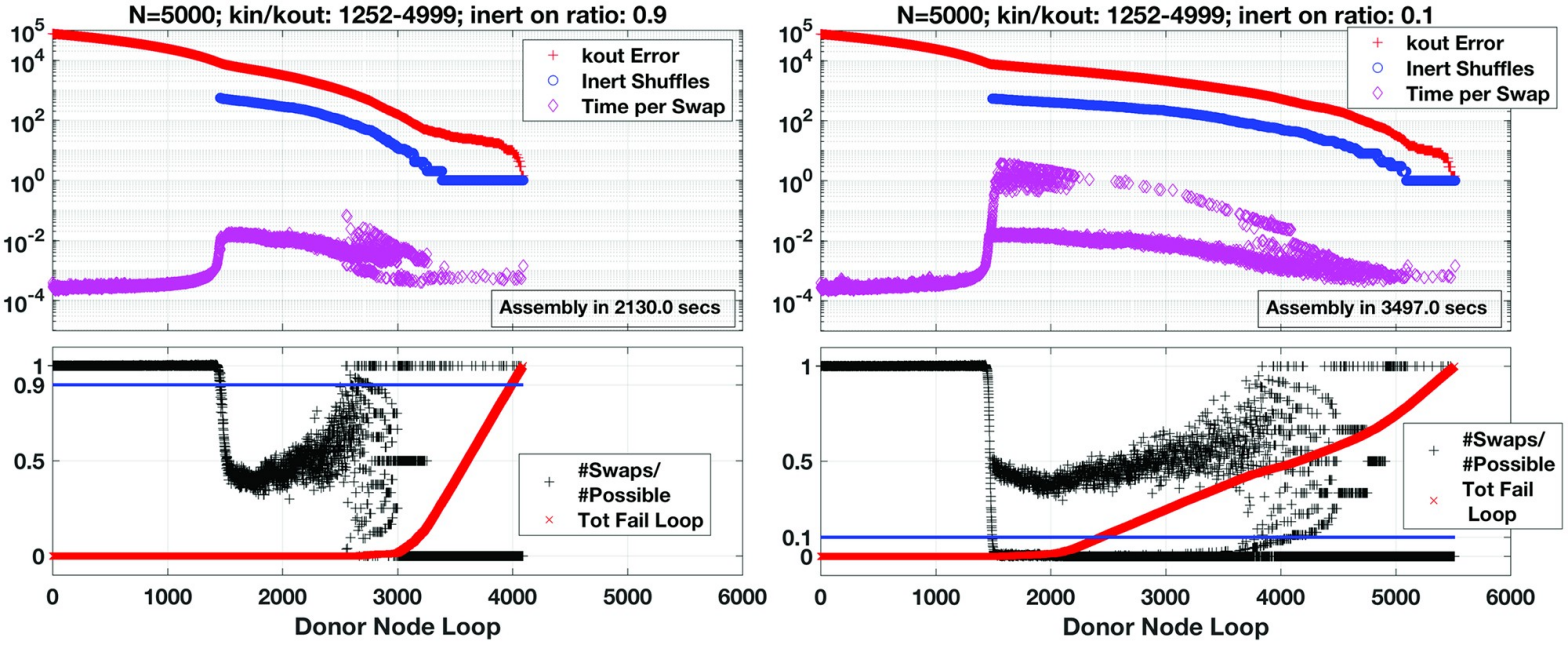
Progression of method reducing error, *r*_*out*_, for sample network sized *N* = 5000; note, degree sequence generated according to a uniform distribution and positive correlation (*ρ* = 0.5) between in- and out-bound degree. This particular sequence further selected for relatively high-density and range of connectivity, with maximal degrees at or near *N* − 1. Error *r*_*out*_ shown over each donor loop, time elapsed per individual edge swap, and number of inert shuffles per loop (upper panes). Note, inert shuffling activated according to two different thresholds: a high activation threshold (left) and low (right), depending on ratio of (# edge swaps / # possible swaps) (black trace, lower panes). Blue traces in lower panes show thresholds of inert shuffling activation for comparison (0.9 and 0.1, respectively). At a high activation ratio of 90%, inert shuffling triggers at donor loop #1457, in this instance, postponing failed loops, defined as donor loops with no edge transfers (normalised total failures over all loops, red trace, lower panes). Compare with the activation ratio set to 10% where inert shuffling enters at donor loop #1495. Although this delay in inert shuffling appears slight, the influence on number of failed loops and also time to perform edge swaps (upper panes) is dramatic, further reducing overall time of assembly by about 40%.

Random selection of a *donor’s* edge to an available *recipient* node in **A** is not always successful. Some scenarios manifest where no open *recipient* edges are available for a given *donor* without violating **k**_**in**_ and the algorithm skips to another *donor* node. If enough of these failed attempts occur, the *inert* nodes make their entrance. We exploit the *inert* nodes as pools of edges for simple randomisation of the established connections of either the *donor* or *recipient* nodes. By randomly swapping edges (non-zero entries of **A**) from the *inert* to either the *donors* or *recipients* such that neither $k_{\text{in}}^{i}$ nor $k_{\text{out}}^{i}$ are affected, we apply a form of simulated annealing to our system—shuffling the network. We activate this *inert* shuffling contingent on the ratio of completed to total possible edge transfers for one loop over all *donor* nodes. For instance, 89 transfers occurring over 100 donor nodes will trigger *inert* shuffling at a threshold of 0.9. Varying this activation threshold can significantly reduce overall workload for the method as illustrated in Fig 3, depending on the network. Earlier activation of the shuffling during assembly effectively increases chances of finding suitable edge transfers reducing overall number of loops over *donors* and hence total time to completion.

#### Demonstration of procedure

For our simple example **A**^(**1**)^ in Eq 5, we can apply the algorithm described and obtain a ‘simple’ graph as shown below in Eq (8). This particular instance of **A**^(**i**)^ required *i* = 9 iterations before *r*_*out*_ fell to zero: our final **A**^(**f**)^ satisfies $k_{\text{out}}^{t}$ exactly. Notably, this demonstration ‘toy’ network does not invoke the *inert* shuffling subroutine, and achieves the goal of matching $k_{\text{out}}^{t}$ without resorting to randomisation of the network connections.

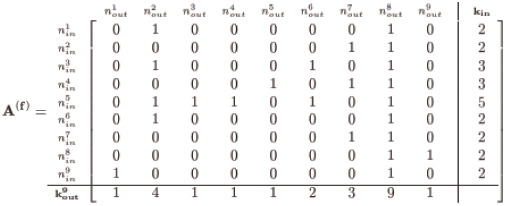
(8)

A quick inspection of the diagonal entries for this **A**^(*f*)^, however, reveals a self-loop for node #8: this is required given the node’s *k*_*out*_ = 9. The algorithm of Fig 2 is easily configurable to permit or deny inclusion of self-loops; and clearly here $k_{\text{out}}^{t}$ requires it. Hence, the designated degree sequences may force inclusion of auto-connections if the degree is equal to the network size; note, subsequent networks shown here are thus with degree sequences less than *N*.

While illustrative, this toy network is not quite comparable to larger sequences found in real-world settings. We thus present examples of the algorithm’s progression for larger networks of size *N* = 5000 in Fig 3, and observe successful ‘convergence’ in both instances after around 4000 and 5500 loops over the *donor* nodes, respectively. Interestingly, all pilot assemblies ranging over *N* = 100, 500, 1000, 2000 and 5000, display the same overall behaviour: rapid initial drop of *r*_*out*_ until exhaustion of available *donor* node edges for transfer to *recipients*. Then very few if any edge transfers occur triggering activation of *inert* node shuffling, or randomising the edge distributions to enable further transfers to *recipient* nodes. This inspired later modifications to the method’s implementation, triggering *inert* shuffling depending on the ratio of actual edge transfers to total possible instead of when no *donor* → *recipient* shifts result. Calibrating the activation of *inert* shuffling leads to the two results shown in Fig 3, where invoking the shuffling subroutine earlier in the method well before exhaustion of available *donor* edges accelerates assembly.

#### Ensuring graphicality

Our implementation of this method includes a preliminary test ensuring the provided sequences of in- and out- degrees may actually realise a graph. We follow the theoretical investigation of [21] with the following definition and relevant theorem.

**Definition 1** *A bidegree sequence* **k** = (**k**_**in**_, **k**_**out**_) *with members* $k_{in},k_{out}\in N\cup 0$, *is graphic if an adjacency matrix*, **A**, *exists with binary entries [0, 1] such that the sum of the i*^*th*^ *row is* $k_{in}^{i}$ *and the j*^*th*^ *column is* $k_{out}^{j}$. *Such an A may have self-loops if the diagonal includes non-zero entries*.

Verification of graphicality for a bidegree sequence **k** is, according to a classic theorem, by virtue of inspecting *N* inequalities.

**Theorem 1** *(Gale-Ryser/Fulkerson [24]) For a bidegree sequence* **k** = (**k**_**in**_, **k**_**out**_) *with* $k_{in}^{i}$ *non-increasing*, **k** *is a graphic sequence if and only if*

$$\begin{array}{c} \sum_{i=1}^{N}k_{in}^{i}=\sum_{i=1}^{N}k_{out}^{i} \end{array}$$ (9)

*and for all j* ∈ [1..*N* − 1]

$$\begin{array}{c} \sum_{i=1}^{N}min\left(k_{out}^{i},j\right)\geq \sum_{i=1}^{j}k_{in}^{i}. \end{array}$$ (10)

For our purposes, we assume a given bidegree sequence **k** passed to the method described above is not sorted in any particular order; but for testing graphicality our implementation pre-processes **k**_**in**_ into a non-increasing sequence. Such a graphicality test is performed only *once* in our implementation; we do not repeatedly inspect resulting adjacency matrices **A**^(*i*)^ during edge transfers unlike the method presented in [25]; hence, successful assembly of any sequence **k** is not guaranteed. In practice, however, we find initial testing of a sequence adequate before launching the permutation assembly and only rarely encounter failures with the method.

#### Multiple edge connections

The permutation method we describe is readily applicable to generating a network given a bidegree sequence **k** and a target percentage of multi-edges in the network. Consider again the toy network illustrated in Fig 1 that includes both self-loops and several multi-edges. Our method may generate such a network with, for instance, the following precursor **A**^(0)^ adjacency matrix with a target multi-edge proportion of around 10%:

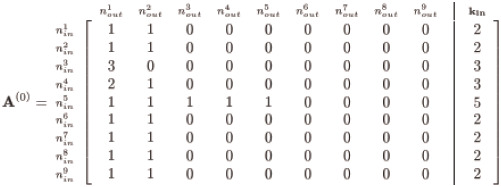
(11)

Multi-edges here we define as connections between nodes *in addition* to existing connections; so, given that definition node 3 has two multi-edges and node 4 one. The process for constructing the precursor **A**^(0)^ assigns so many multi-edges across the nodes such that a given target proportion of multi-edges is approximately met. If otherwise precisely meeting said target violates the given sequences **k**, the method will instead intentionally miss the desired multi-edge proportion since we consider the degrees of nodes inviolate.

**Fig 4:**
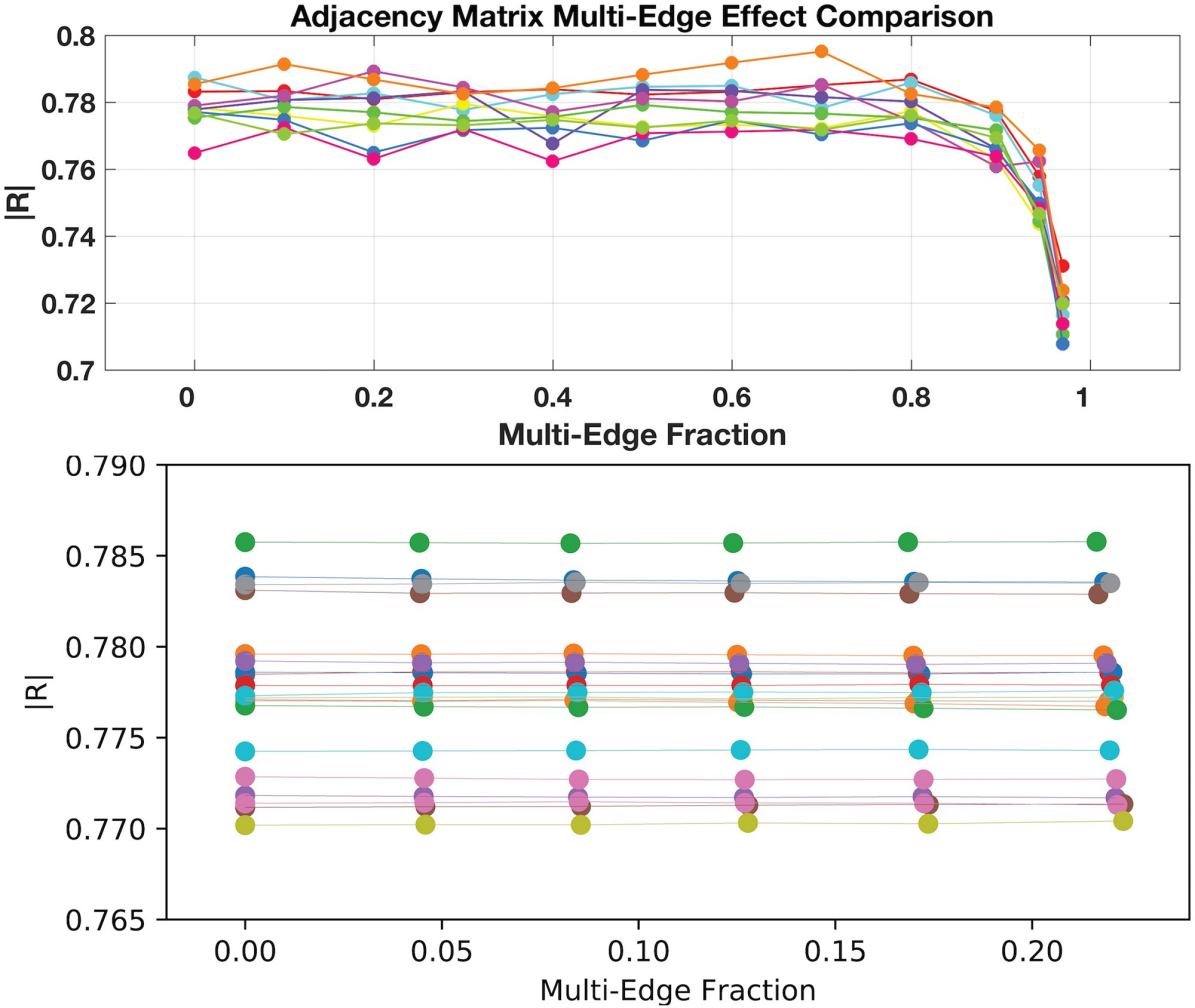
Illustration of multi-edge influence proportion on synchronisation parameter (|*R*|) for neuronal network, sized *N* = 5000. Overall little influence of multi-edges arises until quite high densities occur at around 90% (Upper Pane) as produced by the permutation method. Networks constructed via the Configuration Method (Lower Pane) that arbitrarily generate up to around 20% total multi-edges instead show no effect over range of multi-edges removed via post-processing. Note, the two results presented are via different solution techniques: the upper pane solution of full suite of equations, the lower with a mean-field approximation (see Appendix). Each curve corresponds to a unique realisation of excitability parameter suite for group of *N* neurons in simulation. Generation of individual adjacency matrices, *A*, were performed with degree sequences produced via power-law distribution exhibiting neutral correlation between in- and out- degrees ranging over *k*_*in*_, *k*_*out*_ ∈ [750, 2000]. See Appendix for more details of the neuronal network simulation.

The process of permuting the rows of **A**^(0)^ to an **A**^(1)^ and subsequent edge exchanges from surpluses of *donors* to *recipients* is essentially the same as with solo-edges. However, edge transfers are permitted only between like-connections (multi- to multi- and solo- to solo-) to preserve the proportion of multi-edges along the sequence of **A**^(*i*)^ intermediates. For the example results given, adjacency matrices assembled were expressed with multi-edge proportions as high as 99%. However, our example application to dynamics of a neuronal network was sensitive to resulting assortativities that suffer distortions with multi-edge proportions over around 97%, and we thus restrict those examples within such a threshold. For comparison of the influence on the neuronal dynamics, we present results performed on networks generated with the more traditional Configuration Model combined with multi-edge post-processing removal routines (Fig 4).

Generating suites of the ‘null-model’ spaces corresponding to three real-world networks were performed—comparing the connectivities of *i*) Macaque cortical connectivity [26], *ii*) E. Coli metabolic network [27], and *iii*) Feline cortical and thalamic connectivity [28]. Cases *i*) and *ii*) were with simple networks (i.e., no multi-edges) and compared with generations via the CM. In case *iii*) we varied the multi-edge proportions over a wide range—well beyond the intrinsic ‘weighting’ or proportion of the actual feline network. Although the CM variants include weighted versions, due to its limitations regarding reproducing link densities overall [29], and our interest in considering the influence over a range of multi-edge proportions, we restrict ourselves in case *iii*) to only the method presented in this work.

For inspecting the influence of our method on network characteristics, we compute the average nearest neighbour degree (ANND) similar to other work [30]. Whereas the node degrees are first-order properties depending on the number of links entering and departing a vertex, the ANND is a second-order characteristic describing the paths of length 2, measuring the dependencies between the degrees of neighbouring nodes. This metric provides some insight into the assortativities of networks, a ‘higher order’ characteristic, but permits examination over the range of in- and out- degrees of a network, and is computed thus:

$$\begin{array}{c} k_{i}^{nn}\left(A\right)=\frac{\sum_{j\neq i}\sum_{k\neq j}a_{ij}a_{jk}}{\sum a_{ij}} \end{array}$$ (12)

for a unidirectional network. The analogue for directional networks is given by

$$\begin{array}{c} k_{i}^{nn,out}\left(A\right)=\frac{\sum_{j\neq i}\sum_{k\neq j}a_{ij}a_{jk}}{\sum a_{ij}} \end{array}$$ (13)

$$\begin{array}{c} k_{i}^{nn,in}\left(A\right)=\frac{\sum_{j\neq i}\sum_{k\neq j}a_{ji}a_{kj}}{\sum a_{ji}} \end{array}$$ (14)

and for weighted directional networks

$$\begin{array}{c} k_{i}^{nn,out}\left(A^{*}\right)=\frac{\sum_{j\neq i}\sum_{k\neq j}a_{ij}^{*}a_{jk}^{*}}{A^{*}\sum a_{ij}^{*}} \end{array}$$ (15)

$$\begin{array}{c} k_{i}^{nn,in}\left(A^{*}\right)=\frac{\sum_{j\neq i}\sum_{k\neq j}a_{ji}^{*}a_{kj}^{*}}{A^{*}\sum a_{ji}} \end{array}$$ (16)

where the matrix A* is the weighted analogue of **A** with multi-edges for our purposes here, and a total weighting of A*.

## Results

We present our generated suites of synthetic networks prescribed by given degree sequences for our chosen three datasets of ‘real-world’ systems. The first two are binary simple networks derived from *i*) segregated regions and pathways obtained from anatomical studies of the connections within the cerebral cortex of the macaque [26], and *ii*) the metabolic network of interactions of the common bacterium E. Coli. [27]. The third presented suite is for a weighted—or in our parlance here, including multi-edges—and hence a complex network derived from cortical and thalamic connectivity of a cat (case *iii*) [28]. All sets are bidirectional so we assemble adjacency matrices with given *k*_*in*_ and *k*_*out*_ degree sequences.

**Fig 5:**
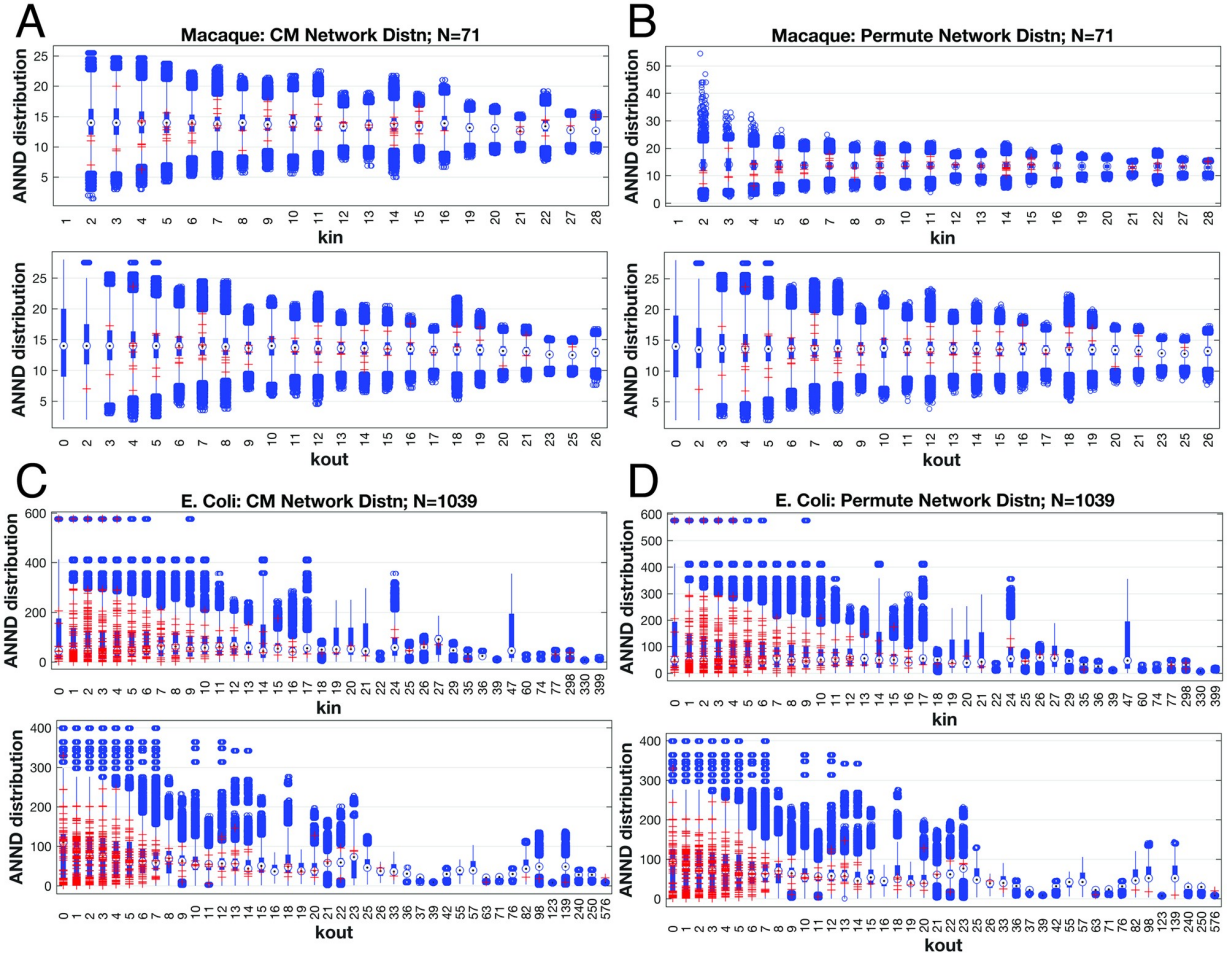
Comparison of null models generated via synthetic network samples with two binary, directed datasets: Macaque cortex (A & B) and metabolic network of E. Coli (C & D). Each dataset provided degree sequences *k*_*in*_ and *k*_*out*_ in turn used to generate *n* = 200, 000 samples of synthetically fabricated networks via the CM (left column) or the permutation method (right column). Whisker plots show ANND values comparing synthetic (blue) and original data (red) values. Means (blue circles), one standard deviation (blue vertical lines) and outliers beyond overall encompass the datasets given for the ANND metric within the outlier bands. The two generation methods show similar distributions overall except for the in-bound degree distribution of the macaque cortex where outliers are generated at significantly higher ANND values for low-degree nodes (Panel B).

We generated *n* = 200, 000 samples of network realisations using both the CM and our permutation method as shown in Fig 5 for each system *i*) and *ii*). Quite similar distributions for these null-models based on the given sequences emerge from both assembly methods for the rather small macaque cortex network (*N* = 71) and the much larger E. coli metabolic network (*N* = 1039). Notably, the actual computed ANND for the raw data generally fall within the distributions—but primarily outside the first quartiles of the whisker plots. It is the outlier groupings—some of which exhibit significant clusters—that encompass the raw data points, suggesting these actual real-world networks that fall well outside of the expected, null-model distributions are indeed not assembled due to random processes.

**Fig 6:**
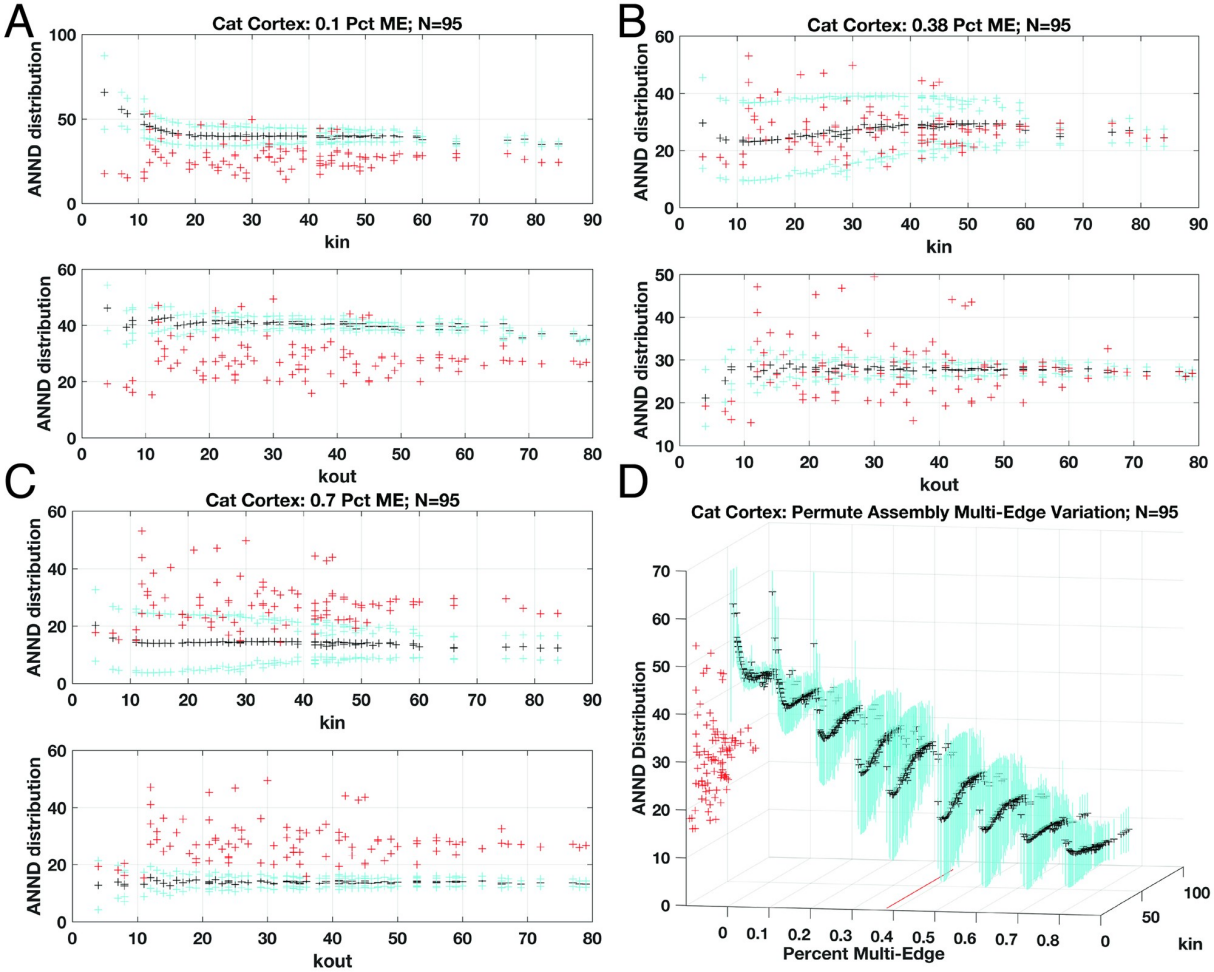
Weighted directional network generations for the cat cortical and thalamus neuronal system with *N* = 95 nodes. Scatter plots show the mean (black), one standard deviation (cyan lines) and original data (red) for ANND computed per in- and out- degrees. Three levels of prescribed multi-edge proportions for null model suites presented for 10%, 38% (as found in raw data), and 70% in Panels A, B and C, respectively. Distributions for *k*_*in*_ ANND values shown over range from no multi-edges (simple network) to 80% in Panel D, with real-network ANND values shown along leftmost plane for comparison. Actual proportion of 38% indicated with red trace at *z* = 0 for comparison. Note significant reduction in ANND values as multi-edge densities increase with asymptotic approach to minimal means, yet standard deviations increase substantially as proportions pass through real-network proportion (cyan vertical traces), then diminish. Note, vertical axis truncates plot for visibility at ANND value of 70 whereas maximal deviations reach up to approximately 100.

We next applied our permutation method to sampling null-model spaces over a wide variety of prescribed densities of multi-edges for a data set derived from the cortical and thalamic regions of the cat. Since the CM technique does not provide a way to control the proportion of multi-edge links [29], we only present synthetic networks produced via our permutation method. The range of multi-edge proportions were set from zero—a simple graph—to 80% for this feline neuronal system sized *N* = 95 (see Fig 6). Rather unsurprisingly, the means and range of standard deviations for the null-models mostly encompass the original ANND metrics when the multi-edge proportion is targeted at the same level of the raw data (Panel B). Over the range displayed, we observe a distinct reduction in the mean value and deviation clouds as multi-edge densities increase that asymptotically approach a rough average mean of around 10 (for *k*_*in*_, Panel D). The out-bound *k*_*out*_ ANND degree distributions—for this particular dataset—are far more confined as can be seen from Panels A-C, and not shown over the full range. Interestingly, the deviation spread is greatest at around the observed intrinsic multi-edge proportions (see distribution along proportion of 0.38) and gradually tightens again upon approach to the maximal multi-edge density assembly performed (distribution along 0.8). Overall, the lower ANND distributions at the higher multi-edge proportions suggests the network exhibits simply fewer low-degree nodes connected to higher-degree nodes, due to concentrations of these multiple edge connected vertices. As the densities of multi-edges falls, the rising of ANND distributions suggest more connections between lower-degree and higher degree nodes: notice the peak values along the simple-graph distribution (percent multi-edge of zero) align with the lowest degree *k*_*in*_.

## Discussion

We applied our method to generation of networks over a variety of sizes and multi-edge densities, and observed the influence of their proportions—very high proportions—on the dynamics of a neuronal network, reducing the overall coherency of a system. Generation of null-model suites for three real-world datasets were further performed, where we showed comparable behaviour between the permutation method presented and the classic CM approach. Applied to realising a null-space encompassing entire ranges of multiple-edge densities, our method illustrated their apparent influence on the statistical alignment of a real-world network within the multi-edge density span.

However, this method we present is not without limitations; particularly regarding the uniform sampling of a graph space. Although the exchange of randomly-selected edge swaps from *donor* to *recipient* nodes along with the so-called *inert* shuffling during the process is essentially a directed MCMC randomisation scheme, it is not clear whether the method introduces bias by guiding the exchanges in such a manner. Noted difficulties of assembling simple graphs with uniform sampling [13] compelled consideration of test examples for inspecting our method’s sampling, as well as a mechanism for providing estimates of weights for the sampling achieved—following the example of [25].

Initially, the use of a randomly permuted **A**^(1)^ appeared to be a possible source of biasing; however, we were fortunate in our use of Matlab’s randperm command. This command *uniformly* permutes integer entries providing an easy start to our analysis here. Subsequent testing of permuted forms for initial **A**^(0)^ → **A**^(1)^ matrices generated for wide ranges of degree sequences and matrix sizes indeed shows uniform distributions throughout (not shown).

**Fig 7:**
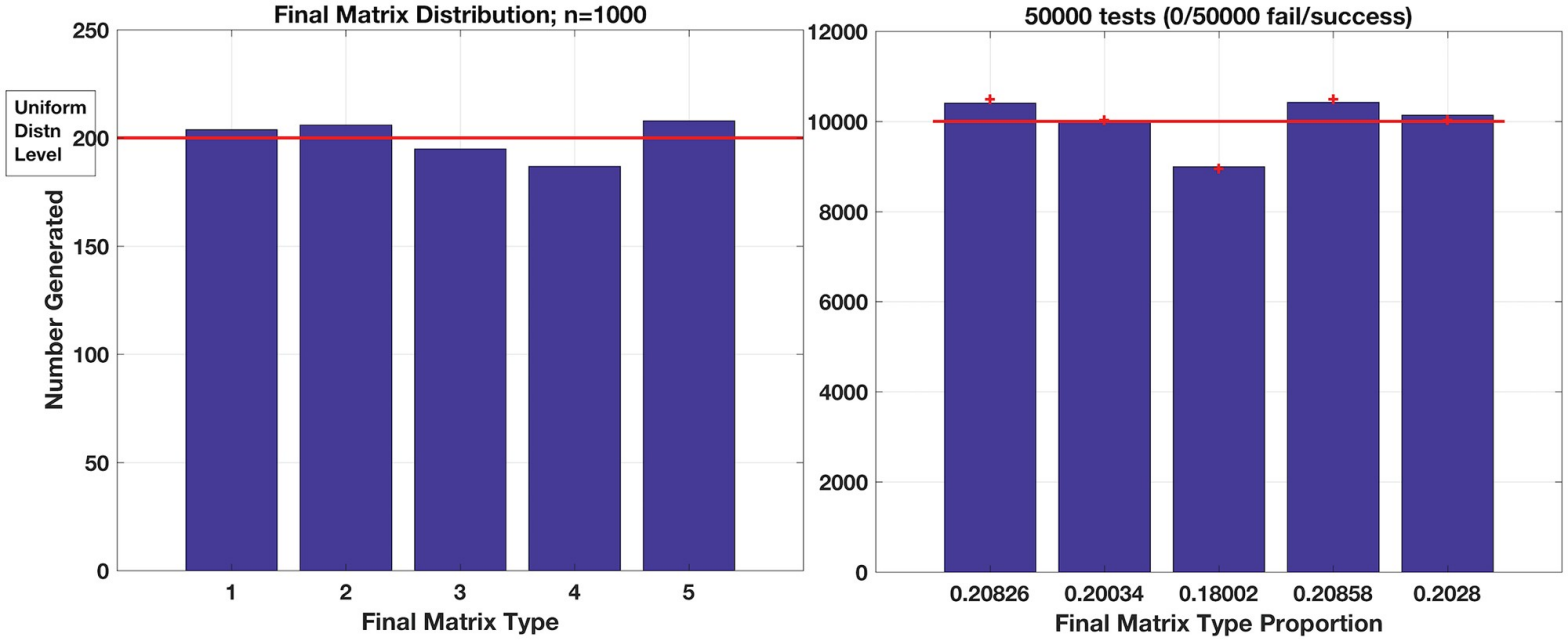
Distribution of resulting 3x3 matrices for predescribed k_in_ and k_out_ = [1 2 1]. The 5 possible results should all emerge with identical probability if the method samples these possibilities uniformly. Testing 1000 matrices (Left Pane), we obtain roughly 1/5 or 20% for each possible matrix **A**^(**f**)^, but only roughly. (Right Pane) Histogram of *n* = 50, 000 samples showing convergence to the expected—and non-uniform—distribution of final **A**^(**f**)^ as calculated through all possible trajectories from **A**^(**0**)^ → **A**^(**f**)^. Red markers above each bar indicates theoretical expected values of 0.2099, 0.2006, 0.1790, 0.2099 and 0.2006 for the 5 types of **A**^(**f**)^, respectively.

On the other hand, the sequence of edge exchanges for intermediate **A**^(*i*)^ enroute to the final satisfactory **A**^(*f*)^ is not trivial. We follow the example investigated in [31] for a simple 3 × 3 case—partly due to the ready tractability of the combinatorics for such a small system. With a **k**_**in**_ and **k**_**out**_ of [1 2 1], only five possible matrices result as follows:

$$\begin{array}{c} A^{\left(f\right)}=(\begin{array}{ccc} 0 & 1 & 0\\ 1 & 0 & 1\\ 0 & 1 & 0 \end{array}),(\begin{array}{ccc} 0 & 1 & 0\\ 1 & 1 & 0\\ 0 & 0 & 1 \end{array}),(\begin{array}{ccc} 1 & 0 & 0\\ 0 & 1 & 1\\ 0 & 1 & 0 \end{array}),(\begin{array}{ccc} 0 & 0 & 1\\ 1 & 1 & 0\\ 0 & 1 & 0 \end{array}),(\begin{array}{ccc} 0 & 1 & 0\\ 0 & 1 & 1\\ 1 & 0 & 0 \end{array}) \end{array}$$ (17)

and notice if you will these resulting adjacency matrices include self-loops, so our discussion here does not concern their exclusion. A suitably uniform sampling of this space should then generate each of these 5 possibilities with even probabilities of simply 1/5 or 20%. We test our method by initialising an **A**^(**0**)^ then randomly permuting entries to generate one of $(\begin{array}{c} 3\\ 1 \end{array})(\begin{array}{c} 3\\ 2 \end{array})(\begin{array}{c} 3\\ 1 \end{array})=27$ possible realisations for **A**^(**1**)^ satisfying **k**_**in**_ but not **k**_**out**_ and generate **A**^(**f**)^ as per our scheme; histograms of the resulting **A**^(**f**)^ production are shown in Fig (7) for two sample sizes. At *n* = 1000, we observe a roughly uniform distribution, yet further sampling does not approach the actual expected 20% proportion for each final type. Hence, we computed the probability for generating each of these **A**^(**f**)^ categories, from initial production of the permuted **A**^(**1**)^ along trajectories of intermediate **A**^(**i**)^ via selected *donor* → *recipient* edge transfers.

This entailed calculating weights of each potential number of edge selections given available pools of *donor* and *recipient* edges. For instance, the following initial **A**^(0)^ (satisfying the prescribed **k**_**in**_), with subsequent permutation into **A**^(1)^ may occur as the following pair,

$$\begin{array}{c} A^{\left(0\right)}=(\begin{array}{ccc} 1 & 0 & 0\\ 1 & 1 & 0\\ 1 & 0 & 0 \end{array}),A^{\left(1\right)}=(\begin{array}{ccc} 1 & 0 & 0\\ 1 & 0 & 1\\ 0 & 0 & 1 \end{array}), \end{array}$$ (18)

that then may follow a few distinct pathways of edge transfers, each with calculable probabilities as the method traverses its way to the final **A**^(*f*)^. For instance, this particular **A**^(1)^ of 18 has two potential transfers from *donor* column number 1 to *recipient* column number 2:

$$\begin{array}{c} A_{a}^{\left(2\right)}=(\begin{array}{ccc} 0 & 1 & 0\\ 1 & 0 & 1\\ 0 & 0 & 1 \end{array}),\text{or},A_{b}^{\left(2\right)}=(\begin{array}{ccc} 1 & 0 & 0\\ 0 & 1 & 1\\ 0 & 0 & 1 \end{array}). \end{array}$$ (19)

that naturally corresponds to a weighting of *w*_1_ = 2, or probability of *p*_1_ = 1/*w*_1_ = 1/2 for either possible **A**^(**2**)^—that we label here with subscripts ‘**a**’ and ‘**b**’. The next edge transfer is then constrained by whether the method traverses through variant $A_{a}^{\left(2\right)}$ or $A_{b}^{\left(2\right)}$, since, if you will notice, the next *donor* column 3 and its two edge rows have either two open sites in the corresponding *recipient* column 2 as with $A_{a}^{\left(2\right)}$, or only one as with $A_{b}^{\left(2\right)}$. Three potential **A**^(**3**)^ matrices then arise:

$$\begin{array}{c} A_{a}^{\left(3\right)}=(\begin{array}{ccc} 0 & 1 & 0\\ 1 & 1 & 0\\ 0 & 0 & 1 \end{array}),\text{or},A_{b}^{\left(3\right)}=(\begin{array}{ccc} 0 & 1 & 0\\ 1 & 0 & 1\\ 0 & 1 & 0 \end{array}),\text{or},A_{c}^{\left(3\right)}=(\begin{array}{ccc} 1 & 0 & 0\\ 0 & 1 & 1\\ 0 & 1 & 0 \end{array}), \end{array}$$ (20)

with $A_{a}^{\left(3\right)}$ and $A_{b}^{\left(3\right)}$ ‘daughter’ matrices emerging from edge transfers of $A_{a}^{\left(2\right)}$. $A_{c}^{\left(3\right)}$ is clearly the only option from $A_{b}^{\left(2\right)}$. The resulting weights for this third step are thus *w*_2*a*_ = 2 and *w*_2*b*_ = 1. Since each of the **A**^(**3**)^ variants are one of the five acceptable **A**^(**f**)^ satisfying the prescribed bidegree sequence **k**, the method halts here. This gives three weights per **A**^(**1**)^ → **A**^(**3**)^ path as follows. $A^{\left(1\right)}\to A_{a}^{\left(2\right)}\to \left(A_{a}^{\left(3\right)}\;\text{or}\;A_{b}^{\left(3\right)}\right)$ corresponds to *w*_1_ × *w*_2*a*_ = 2 × 2 = 4, and $A^{\left(1\right)}\to A_{b}^{\left(2\right)}\to A_{c}^{\left(3\right)}$ corresponds to *w*_1_ × *w*_2*b*_ = 2 × 1 = 2. The probabilities for each three outcomes are simply then $p_{a}^{3}=p_{b}^{3}=1/4$ and $p_{c}^{3}=1/2$, and we observe such proportions of 25:25:50% for this suite of **A**^(**1**)^ → **A**^(*f*)^ pathways.

Calculating the weights and probabilities over each individual trajectory from the 27 possible **A**^(**1**)^ initial permutations to the five acceptable **A**^(**f**)^ presents the expected distribution of matrix generation as illustrated in Fig 7b. The histogram of **A**^(**f**)^ production shows convergence to the calculated probabilities after *n* = 50, 000 tests.

**Fig 8:**
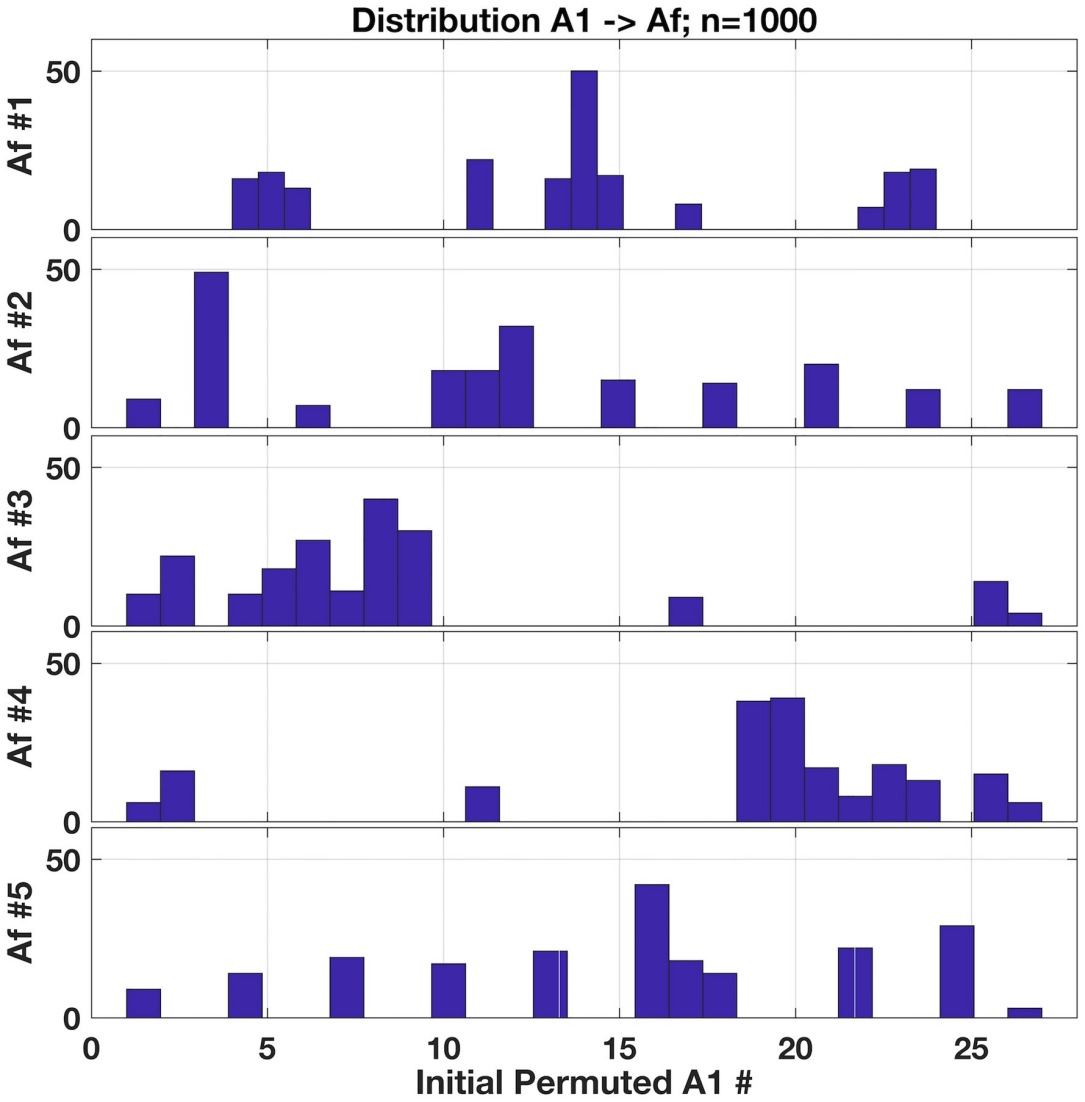
Distributions of five possible A^(f)^ outcomes as produced by initial, permuted A^(1)^. Each pane shows a histogram of the 27 **A**^(**1**)^ that eventually lead to the **A**^(**f**)^ as noted, where distinctive patterns emerge. Observe a roughly tri-banded distribution for $A_{1}^{\left(f\right)}$ and a complementary banding between $A_{3}^{\left(f\right)}$ and $A_{4}^{\left(f\right)}$. Note how most initial **A**^(**1**)^’s numbered between 1 and 10 lead to $A_{3}^{\left(f\right)}$ whereas initial **A**^(**1**)^’s numbered roughly from 20 to 27 lead to $A_{4}^{\left(f\right)}$.

The deviation we observe here from the ideal uniform distribution as shown in Fig (7) is apparently due to the restriction of trajectories from an initial, permuted **A**^(**1**)^ to subsets of all possible **A**^(**f**)^—as illustrated in our example calculation for the trajectory weights above. Distributions of **A**^(**1**)^ → **A**^(**f**)^ realisations are shown in Fig (8) where we clearly see the **A**^(**f**)^ depend on which initial permutation launches the procedure. The method’s permutation of **A**^(**0**)^ into a **A**^(**1**)^—albeit uniformly—nevertheless restricts potential **A**^(**f**)^ outcomes: the set of permitted *donor* → *recipient* edge exchanges is clearly shaped by the initial condition, if you will, established by a given **A**^(**1**)^.

Clearly, the method does not uniformly sample the graph space. We, however, follow the presentation given in [25] for utilising the weights of the sampling as already illustrated for our scheme’s method traversing pathways from **A**^(**1**)^ → **A**^(**f**)^. Given these weights, we can deploy a well-known biased sampling result [32], and calculate the weighted average for our suite of **A**^(**f**)^ via

$$\begin{array}{cc} \left\langle Q_{s}\right\rangle & =\frac{\sum_{j=1}^{M}w\left(t_{j}\right)Q\left(t_{j}\right)}{\sum_{j=1}^{M}w\left(t_{j}\right)} \end{array}$$ (21)

for some metric *Q*. We here treat the quantity *Q* as the spectral radius for the resulting **A**^(**f**)^. Kim, et al., [25] used the assortativities of the adjacency matrix; however, for our tractable 3 × 3 example, the assortativities of the **A**^(**f**)^ shown in Eq 17 are mostly zero distorting the sample mean calculation. *Q*_*s*_ is here simply the ‘sampled’ spectral radius over all possible pathway realisations for *M* samples of the trajectory space; of course, as *M* → ∞ the sample mean should approach the actual arithmetic mean. Each **t**_**j**_ we consider the *j*^*th*^ trajectory from **A**^(**1**)^ → **A**^(**f**)^ sampled, and *w*(**t**_**j**_) the weight resulting from the sequence of available edge transfers from *donor* → *recipient* nodes during the method’s traversal through this trajectory space, readily calculated via

$$\begin{array}{c} w\left(t\right)=\prod_{i}\prod_{j=1}^{n_{e}}d_{i}\left(j\right). \end{array}$$ (22)

The inner product is over the number of available edges (*n*_*e*_) for *donor* (*d*_*i*_) → *recipient* exchange, and the outer product is over the intermediate adjacency matrices, **A**^(**i**)^ along the way as illustrated in our example calculation above.

**Fig 9:**
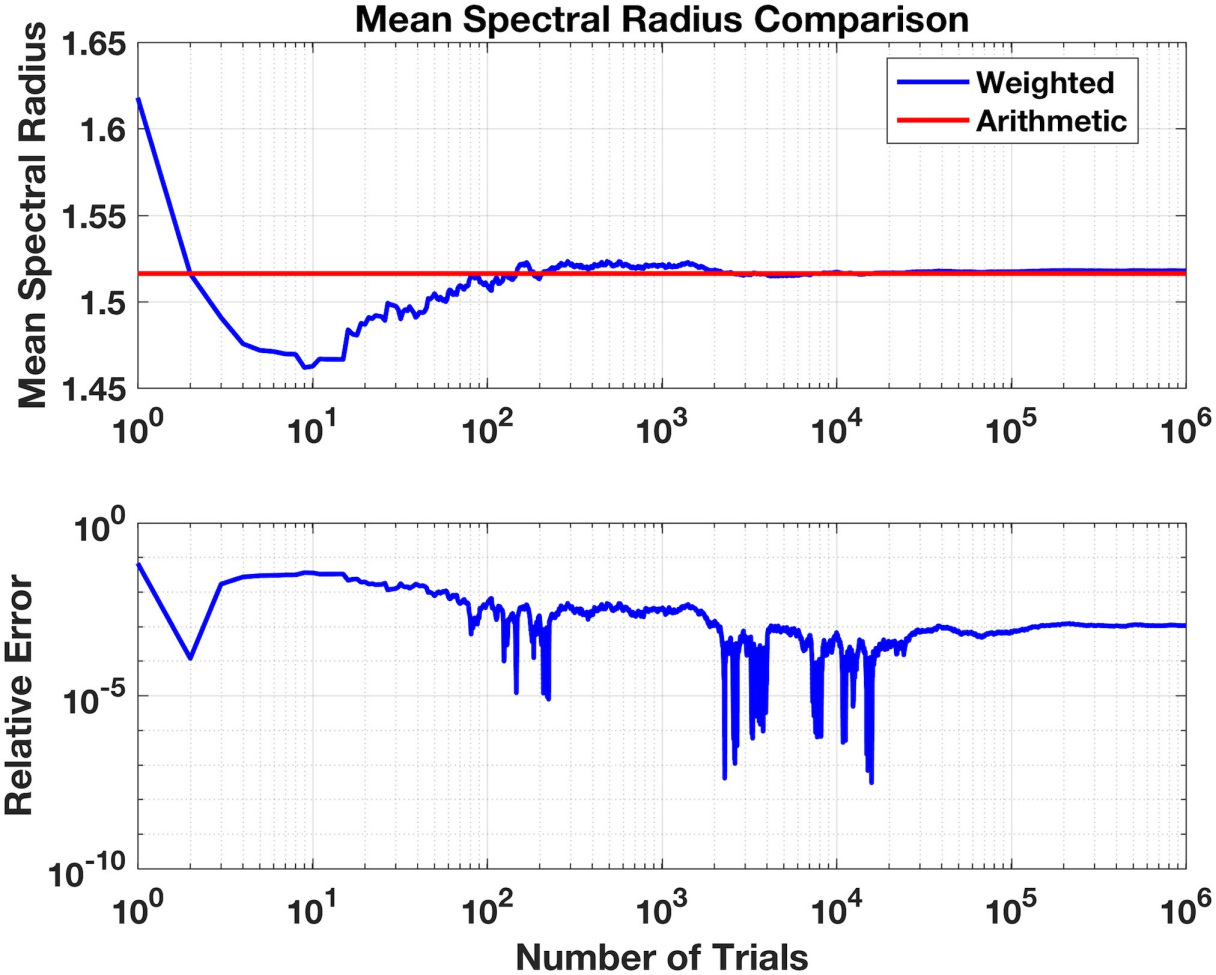
Comparison arithmetic average spectral radius over all five possible realisations for bi-degree sequence k = [1 2 1] and sampled mean spectral radius with weights via permutation method (see text).

Each spectral radius for the matrices in Eq 17 is straightforward enough to compute and are 1.6180, 1.6180, 1.4142, 1.4656, and 1.4656, for the five **A**^(**f**)^, respectively. With an arithmetic mean of 〈*Q*_*a*_〉 = 1.51628, we should observe the weighted sampling mean 〈*Q*_*s*_〉 of Eq 21 approach this value given large enough sample size, *M*; results are shown in Fig 9. Over sampling of *M* = 10^6^, we see indeed the sampled average of spectral radius over the trajectories readily approach the arithmetic with relative errors falling below *O*(10^−5^) but then settling at around *O*(10^−3^). We thus demonstrate how a metric of interest, here the spectral radius, may be accurately estimated over a cohort of network samples by these weighted estimates.

Weights of these samplings are provided in our MATLAB implementation and are rather straightforward to substitute for some *Q* of interest—they are not limited to only the spectral radius. The hazard of combinatorial explosion requires accommodation, however. This example matrix is quite manageable yet larger and more relevant networks demand, for instance, handling weights with symbolic-valued type variables—otherwise overflow of double-valued variables is certain. Nevertheless, if estimates of a metric *Q* are required for some sampling of a network regime, these weights provide the means to find an accurate representation.

Direct performance comparisons with other methods are not presented here, although we do obtain networks up to *O*(10^4^) on the scale of minutes with a laptop running recent versions of Matlab—depending on the degree sequences. The majority of networks we utilised for simulations of neuronal dynamics were sized *N* = 5000 and hence more challenging to assemble than the real-world network comparisons with *N* of 1000 nodes or less. Assembly difficulty depended on the degree sequence and hence edge-density of the network; for the neuronal studies we utilised degree sequences ranging over **k**_**in**_, **k**_**out**_ ∈ [750–2000], and assembly completed all within around 2 minutes for any multi-edge proportion (see Table 1). Times to assemble are primarily dependent on initial distance from target, $r_{out}^{0}$. Networks with highly-dense edge counts (e.g., *N* = 10, 000, **k** ∈ [8500–9900]) still complete assembly quite rapidly compared to lower edge-density networks—that exhibits greater initial $r_{out}^{0}$ by an order of magnitude. The initial error $r_{out}^{0}$ is apparently driven higher with minimum and maximum degrees spread rather widely over the network, as in the last two entries of Table 1 with *N* = 10, 000 and **k** spanning 3000-9900. These wide distributions of in- and out- degrees typically require substantially more time to assemble, although notably earlier activation of *inert* shuffling mitigates this (also see Fig 3). Note, most assemblies shown in Table 1 reported zero *inert* shuffles; this was not due to the ratio of activation set at 0.5. We tested the same **k** at higher shuffling activation ratios to no avail: these particular sequences simply do not require any *inert* shuffling to complete assembly, in contrast with other networks that fail without it.

**Table 1:** Performance statistics.

*N*	k range	Edges	$r_{out}^{0}$	Swaps	Shuffles	Time	Notes
5,000	750-2000	5.4e6	2.2e4	6.2e5	0	134 secs	No multi
5,000	750-2000	5.4e6	2.2e4	6.2e5	0	139 secs	50% multi
5,000	750-2000	5.4e6	2.2e4	6.2e5	0	136 secs	90% multi
10,000	8500-9900	9.2e7	4.0e4	1.7e6	0	7.8 mins	Inert on 0.5
10,000	1500-4000	2.8e7	7.2e4	3.1e6	0	14.2 mins	Inert on 0.5
10,000	2000-7500	4.8e7	1.6e5	6.8e6	0	34 mins	Inert on 0.5
10,000	3000-9900	6.5e7	2.0e5	8.6e6	4.1e5	3.2 hrs	Inert on 0.5
10,000	3000-9900	6.5e7	2.0e5	8.6e6	4.2e5	2.8 hrs	Inert on 0.9

A brief note on successfulness of the method. As mentioned above, we only test for graphicality of **k** given at the outset, and do not persist in testing the resulting intermediate **A**^(*i*)^ during assembly—in contrast to other methods as in [25]. Interim phases of our procedure ignores potential violations of ‘Star-constrained graphicality’ or whether a current edge addition will break graphicality of the system [33]. Instead, the effort at transferring edges maintaining **k**_**in**_ aimed at improving approximation to the target **k**_**out**_ appears to avoid this issue altogether—mostly. We do observe failed assembly attempts with smaller-sized networks (e.g., *N* = 5) that do not engage *inert* shuffles (not shown). Although the edge transfers of the method are valid, it nevertheless encounters a *cul-de-sac* where no subsequent transfers are possible. Such test suites however show success rates of around 80% or better depending on the initial permutation, **A**^(1)^. For our purposes with *N* ranging from the real-world networks sized *O*(10) to substantially larger for the neuronal study, we observe few if any failed assemblies—if *inert* shuffling is enabled. Without the shuffling of edges, the method typically stalls once the reservoir of *donor* surplus edges aligned with *recipient* nodes is exhausted, as illustrated in Fig 3 when the numbers of *donor* swaps fall to zero.

In contrast to myriad assembly methods available, our scheme permits control over proportions of multi-edges in the final assembly while meeting a prescribed degree sequence exactly. However, if matching the given degree sequence is not necessary, then the Chung-Lu method [10] that rapidly pulls adjacency matrices based on probabilities of edge formations is faster than our method presented here. Although Chung-Lu does not meet the exact degree sequence, it does match the expectation and for some null-model comparisons this is perfectly suitable. This was not the case for our study of neuronal network dynamics; moreover, the Chung-Lu method provides no control over multi-edge proportions and was partly inspirational for the formation of our permutation method. Alternatively, an extension of the Chung-Lu method that exploits hypergeometric distributions for assignment of edges [11] satisfies the expectation of node degree but also generates networks including multi-edges. Yet, it does so without direct control over their density as with our permutation method that also meets the degree sequences exactly—albeit not as quickly as the Chung-Lu technique. The ‘soft’ CM as described in [12] alternatively aims at meeting the degree distributions instead of the exact sequence. This nicely accommodates real-world networks exhibiting scale-free power-law distributions and particularly with fluid vertex edge counts—instead of the static degree assignments typically analysed as we have done here. Given the lack of control over multi-edges in the CM, however, we were compelled to formulate an alternative technique that provides such control for determination of their importance in the dynamics of neuronal networks—and illustrated here as a null-model generation method permitting exploration of the space of multi-edge densities.

We have presented our scheme for assembly of directed networks given a bi-degree sequence, into a crowded arena of generation methods with varied strengths and weaknesses. This scheme permits exclusion or inclusion of multi-edges and self-loops, and allows prescribing the proportion of multi-edges in the resulting network unlike other methods. It further meets exactly a given degree sequence, yet it is limited in that the method does not uniformly sample the resulting graph space. Nevertheless, computing the weighted samples does converge to expected means for the adjacency metric given enough samples for all possible trajectories. Overall, we have further found the method quite successful at completing assemblies but cannot guarantee that any sequence initially satisfying graphicality will indeed produce a network. Further analysis of the apparent dependence on randomisation of *inert* shuffling for success and whether the method as described inadvertently respects graphicality constraints during construction may illuminate these issues. Nevertheless, this novel permutation method supplies a means to generate networks with control over multi-edge appearances, providing another tool among a spectrum of network assembly techniques [14, 25, 34–42] meeting either expectations, distributions or exact values of degree sequences—none of which, to our knowledge, permits such control.

## Appendix

**Fig 10:**
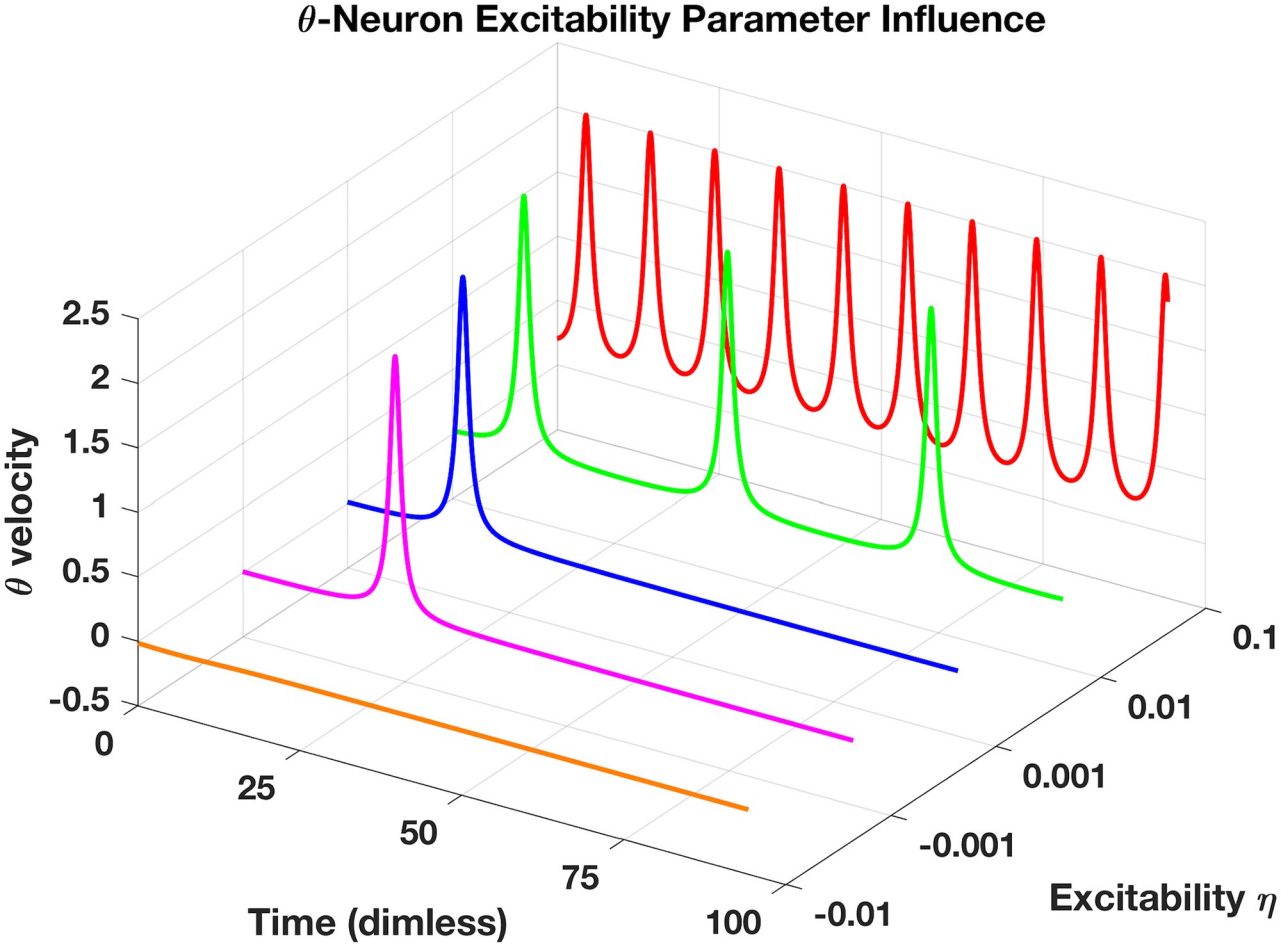
Illustration of *θ*-neuron behaviour for solo neuron simulations with no input (*I* = 0) over range of intrinsic excitability parameter, *η*, showing idealised action-potentials when velocity of *θ*-neuron spikes. For positive *η*, *θ*-neuron oscillates at increasing frequency from *η* = 0.01 to 0.1, spikes only once within this timeframe for 0 < *η* < 0.01, or simply settles to equilibrium if *η* is negative.

A brief description of the neuronal network model is presented here; for further detail, please see our companion paper [7]. We utilise the Theta Neuron model [43] to simulate a network of *N* spiking neurons

$$\begin{array}{c} \frac{d\theta_{i}}{dt}=\left(1-cos\left(\theta_{i}\right)\right)+\left(1+cos\left(\theta_{i}\right)\right)\cdot \left(\eta_{i}+I_{i}\right) \end{array}$$ (23)

where *i* ∈ [1, *N*]. While the state variable *θ*_*i*_ has no physical expression, neuron *i* is said to fire at *θ*_*i*_ = *π*. A neuron’s firing rate is determined by an intrinsic parameter *η*_*i*_ and an external stimulus *I*_*i*_. In the absence of *I*_*i*_ a neuron’s dynamics undergoes a saddle-node bifurcation on an invariant circle (SNIC) as *η*_*i*_ is varied through 0, i.e. for *η*_*i*_ < 0 the state *θ*_*i*_ rests at a stable fixed point whereas for *η*_*i*_ > 0 it is in a stable periodic orbit (see Fig 10) As the firing frequency can become arbitrary low the Theta Neuron is a model for a Type I neuron. In contrast, a Type II neuron exhibits a finite minimal firing rate. Note, that each neuron *i* has a designated *η*_*i*_, thus we model a network of heterogeneous neurons.

The current *I*_*i*_ is composed of synaptic pulses *P*_*n*_(*θ*_*j*_) of inward connected neurons *j* as defined in the adjacency matrix *A*_*i*,*j*_

$$\begin{array}{c} I_{i}=\kappa \cdot \frac{1}{\left\langle k\right\rangle }\sum_{j=1}^{N}A_{i,j}P_{n}\left(\theta_{j}\right) \end{array}$$ (24)

where 〈*k*〉 is the mean degree of the network and *κ* the coupling strength. To model a single pulse-like current we let

$$\begin{array}{c} P_{n}\left(\theta_{j}\right)=d_{n}{\left(1-cos\left(\theta_{j}\right)\right)}^{n} \end{array}$$ (25)

$$\begin{array}{c} d_{n}:\int_{0}^{2\pi }P_{n}\left(\theta_{j}\right)=2\pi \end{array}$$ (26)

with *d*_*n*_ calibrated such that the integral of *P*_*n*_ is independent of *n*, a parameter which is associated with the pulse’s sharpness. Further we find that *max*(*P*_*n*_) is at *θ* = *π* and in the limit *n* → ∞ Eq 25 becomes a delta function. Notice the pulse function *P*_*n*_ is treated as identical across our suite of Theta Neurons: all are shaped to the same order *n*, yet fire independently.

The coupling itself is captured in the adjacency matrix *A*_*i*,*j*_ which is of particular interest here, since it is the matrix of connections between neurons. Connections are considered unidirectional in the networks; i.e., no explicit feedback mechanism from neuron *i* back to neuron *j*, so all influences are exerted downstream. Entries of *A*_*i*,*j*_ are with integer terms only: *a*_*i*,*j*_ = 1 if connected, zero if not, unless they exhibit ‘multi-edge’ connections, then *a*_*i*,*j*_ > 1. Connectivity of a neuron and the number of incoming impulses from upstream neurons, or its input-degree, is thus via summation over *A*_*i*,*j*_ with a fixed *i*:

$$\begin{array}{c} k_{i}^{in}=\sum_{j=1}^{N}A_{i,j}\;\text{and}\;\text{analogous}\;k_{j}^{out}=\sum_{i=1}^{N}A_{i,j}. \end{array}$$ (27)

The mean degree 〈*k*〉 can be computed as the mean value of either *k*^*in*^ or *k*^*out*^

$$\begin{array}{c} \left\langle k\right\rangle =\frac{1}{N}\sum_{i=1}^{N}k_{i}^{in}=\frac{1}{N}\sum_{i=1}^{N}\sum_{j=1}^{N}A_{i,j}. \end{array}$$ (28)

In order to compare network dynamics of the respective adjacency matrix *A*_*i*,*j*_ we consider the coherency of the network, or how synchronised the suite of neurons is at any time. The Kuramoto order parameter, *R*, is computed as an average over all the states thus:

$$\begin{array}{c} R\left(t\right)=\frac{1}{N}\sum_{j=1}^{N}e^{i\theta_{j}}. \end{array}$$ (29)

**Fig 11:**
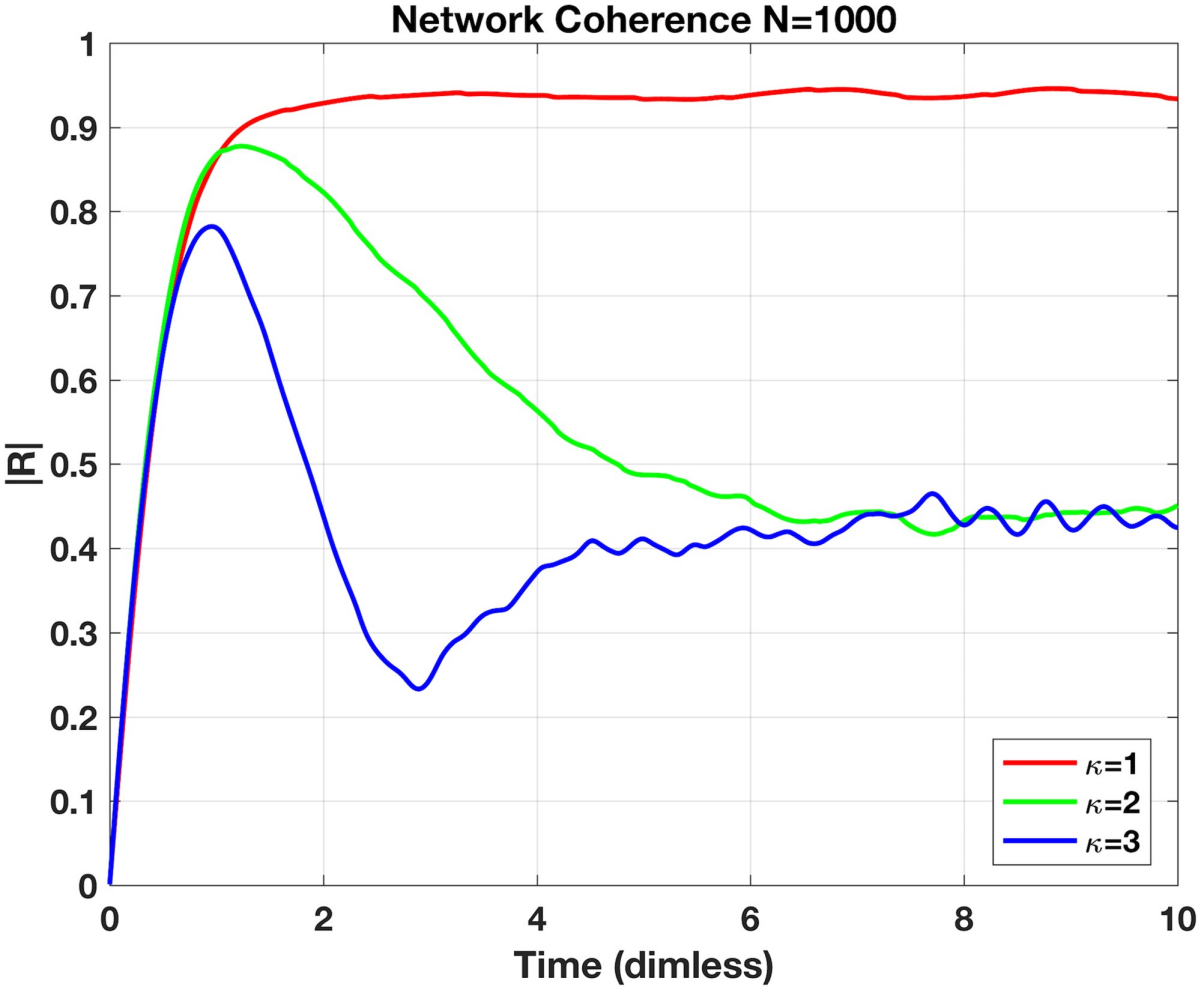
Magnitude of network coherency parameter, |*R*|, plotted over dimensionless time for network sized *N* = 1000. Each trace with excitability parameters, *η* drawn from Cauchy distribution with *η*_0_ = −2, Δ = 0.1, and three different coupling strengths, *κ* = 1, 2 or 3. Connectivity of system, *A*_*ij*_, with ‘neighbour-to-neighbour’ connections; e.g., *n*_1_ → *n*_2_ → *n*_3_…*n*_1000_ → *n*_1_. Initial state of network set to minimal coherency (|*R*| = 0) via even distribution of *θ*_0_’s over unit circle. Each network settles into steady-state coherency of either roughy 50% (green and blue traces) or nearly full coherency (red trace). The lowest coupling strength here, *κ* = 1, rather paradoxically leading to higher coherence is due to overall quiescence of individual neurons. The suite of excitability parameters, *η*, are mostly negative and the small proportion of excitable neurons (*η* > 0) restrained by the diminished coupling cannot stimulate their resistant neighbours to spiking—hence the overall network coherency is quite high.

This metric is complex-valued and tracks both magnitude and angle providing the degree of network synchronisation (|*R*| ∈ [0, 1] with 1 highly synchronised) and the overall *θ* for the network. An illustration of this metric is given in Fig 11 for a small network of *N* = 10.

The presented results of the coherency parameter, *R*, in Fig 4A were performed with a full simulation of the *N* = 5000 discrete *θ*-neuron equations in Eq 23, with a Cauchy-distribution of excitability *η* parameters set to the following: *η*_0_ = −2 (center of distribution) and Δ = 0.1 (width of distribution). The strength of downstream impulses, *κ*, was set to 3 and the sharpness of the pulse function of Eq 25 with *n* = 2. Alternatively, the results given in Fig 4B were performed with a ‘mean-field’ approximation to the full discrete *θ* network; see our companion paper in [7] for details of that approach.

## Supporting information

S1 File (PDF)
