# Supplementary material for: A permutation method for network assembly
Source: PLoS One. 2020 Oct 23;15(10):e0240888. doi: 10.1371/journal.pone.0240888 (PMC7584203; doi:10.1371/journal.pone.0240888)
Supplement: S1 File — (PDF) [file pone.0240888.s001.pdf]

DEGREE ASSORTATIVITY IN NETWORKS OF SPIKING NEURONS

CHRISTIAN BLÄSCHE, SHAWN MEANS, AND CARLO R. LAING

Abstract. Degree assortativity refers to the increased or decreased probability of connecting two neurons based on their in- or out-degrees, relative to what would be expected by chance. We investigate the effects of such assortativity in a network of theta neurons. The Ott/Antonsen ansatz is used to derive equations for the expected state of each neuron, and these equations are then coarse-grained in degree space. We generate families of effective connectivity matrices parametrised by assortativity coefficient and use SVD decompositions of these to efficiently perform numerical bifurcation analysis of the coarse-grained equations. We find that of the four possible types of degree assortativity, two have no effect on the networks' dynamics, while the other two can have a significant effect.

1. Introduction

The effects of the structure of a network of neurons on its dynamics is a large topic of current interest [35, 17, 21, 29, 30, 31, 19]. Here we consider the effects of degree assortativity in a large network of theta neurons. Assortativity in this context refers to the probability that a neuron with a given in- and out-degree connects to another neuron with a given in- and out-degree. If this probability is what one would expect by chance, given the neurons' degrees, the network is referred to as neutral. If the probability is higher (lower) than one would expect by chance the network is assortative (disassortative).

Assortativity has often been studied in undirected networks, where a node simply has a degree, rather than in- and out-degrees (the number of connections to and from a node, respectively) [28, 25, 24]. Since neurons form *directed* connections, there are four types of assortativity to consider [10]: between either the in- or out-degree of a presynaptic neuron, and either the in- or out-degree of a postsynaptic neuron (Figure 1). We are aware of only a small number of previous studies in this area [30, 13]. Kähne et al. [13] considered networks with equal in- and out-degrees and investigated degree assortativity, effectively correlating both in- and out-degrees of pre- and post-synaptic neurons. They mostly considered networks with discrete time and a Heaviside firing rate, i.e. a McCulloch-Pitts model [22]. They found that positive assortativity created new fixed points of the model dynamics. Schmeltzer et al. [30] also consider networks with equal in- and out-degrees and investigated degree assortativity. These authors considered leaky integrate-and-fire neurons and derived approximate self-consistency equations governing the steady state neuron firing rates. They found, among other things, that positive assortativity increased the firing rates of high-degree neurons and decreased that of low-degree ones. Positive assortativity also seemed to make the network more capable of sustained activity when the external input to the network was low.

Date: October 4, 2019.

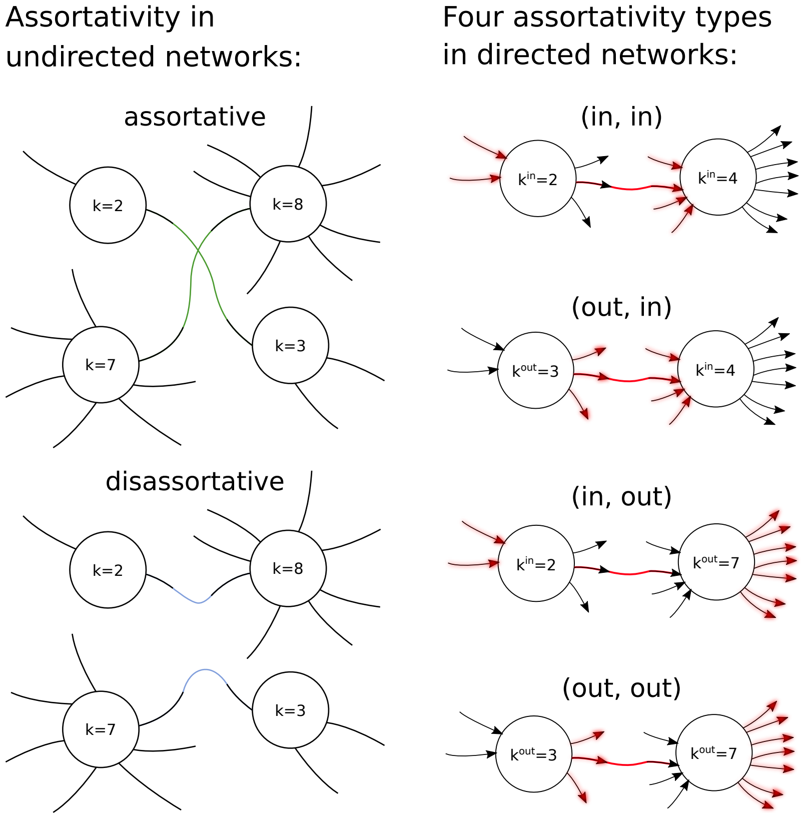

Figure 1. Assortativity in undirected and directed networks. An undirected network (left column) is assortative if high degree nodes are more likely to be connected to high degree nodes, and low to low, than by chance (top left). Such a network is disassortative if the opposite occurs (bottom left). In directed networks (right column) there are four possible kinds of assortativity. The probability of a connection (red) is thus influenced by the number of red shaded links of the sending (left) and receiving (right) node.

Experimental evidence for positive degree assortativity includes [6], who examined a neuronal culture but did not determine directionality of connections. Eguíluz et al. [7] also found evidence of positive assortativity in the brain, and did not determine directionality of connections. They also found evidence for a power law degree distribution, as we consider here.

The outline of the paper is as follows. In Sec. 2 we present the model and then derive several approximate descriptions of its dynamics. In Sec. 3 we describe the method for creating networks with prescribed types of degree assortativity, and in Sec. 4 we discuss aspects of the numerical implementation of the reduced model. Results are given in Sec. 5 and we conclude with a discussion in Sec. 6. Appendix A contains the algorithms we use to generate networks with prescribed assortativity.

2. Model description and simplifications

We consider a network of N theta neurons:

$$\frac{d\theta_j}{dt} = 1 - \cos\theta_j + (1 + \cos\theta_j)(\eta_j + I_j) \tag{1}$$

for $j = 1, 2, \dots N$ where

$$I_j = \frac{K}{\langle k \rangle} \sum_{n=1}^{N} A_{jn} P_q(\theta_n) \tag{2}$$

η_j is a constant current entering the jth neuron, randomly chosen from a distribution $g(\eta)$, K is strength of coupling, $\langle k \rangle$ is mean degree of the network, and the connectivity of the network is given by the adjacency matrix A, where $A_{jn} = 1$ if neuron n connects to neuron j, and zero otherwise. The connections within the network are either all excitatory (if $K > 0$) or inhibitory (if $K < 0$). Thus we do not consider the more realistic and general case of a connected population of both excitatory and inhibitory neurons, although it would be possible using the framework below.

The theta neuron is the normal form of a Type I neuron which undergoes a SNIC bifurcation as the input current is increased throught zero [8, 9]. A neuron is said to fire when θ increases through π, and the function

$$P_q(\theta) = a_q(1 - \cos\theta)^q; \qquad q \in \{2, 3, \dots\} \tag{3}$$

in (2) is meant to mimic the current pulse injected from neuron n to any postsynaptic neurons when neuron n fires. a_q is a normalisation constant such that $\int_0^{2\pi} P_q(\theta)d\theta = 2\pi$ independent of q.

The in-degree of neuron j is defined as the number of neurons which connect to it, i.e.

$$k_j^{in} = \sum_{n=1}^{N} A_{jn} \tag{4}$$

while the out-degree of neuron n is the number of neurons it connects to, i.e.

$$k_n^{out} = \sum_{j=1}^{N} A_{jn} \tag{5}$$

Since each edge connects two neurons, we can define the mean degree

$$\langle k \rangle = \frac{1}{N} \sum_{j=1}^{N} k_j^{in} = \frac{1}{N} \sum_{n=1}^{N} k_n^{out} = \frac{1}{N} \sum_{j=1}^{N} \sum_{n=1}^{N} A_{jn} \tag{6}$$

Networks such as (1) have been studied by others [20, 2, 14], and note that under the transformation $V = \tan(\theta/2)$ the theta neuron becomes the quadratic integrate-and-fire neuron with infinite thresholds [23, 18].

2.1. An infinite ensemble.

As a first step we consider an infinite ensemble of networks with the same connectivity, i.e. the same A_{jn}, but in each member of the ensemble, the value of η_j associated with the jth neuron is randomly chosen from the distribution

$g(\eta)$ [1]. Thus we expect a randomly chosen member of the ensemble to have values of η in the ranges

$$\begin{aligned} \eta_1 &\in [\eta_1', \eta_1' + d\eta_1'] \\ \eta_2 &\in [\eta_2', \eta_2' + d\eta_2'] \\ &\vdots \\ \eta_N &\in [\eta_N', \eta_N' + d\eta_N'] \end{aligned} \tag{7}$$

with probability $g(\eta_1')g(\eta_2')\dots g(\eta_N')d\eta_1' d\eta_2' \dots d\eta_N'$. The state of this member of the ensemble is described by the probability density

$$f(\theta_1, \theta_2, \dots, \theta_N; \eta_1, \eta_2, \dots \eta_N; t) \tag{8}$$

which satisfies the continuity equation

$$\frac{\partial f}{\partial t} = -\sum_{j=1}^{N} \frac{\partial}{\partial \theta_j} \left\{ \left[1 - \cos\theta_j + (1 + \cos\theta_j)(\eta_j + I_j) \right] f) \right\} \tag{9}$$

If we define the marginal distribution for the jth neuron as

$$f_j(\theta_j, \eta_j, t) = \int f(\theta_1, \theta_2, \dots \theta_N; \eta_1, \eta_2, \dots \eta_N; t) \prod_{k \neq j} d\theta_k d\eta_k \tag{10}$$

we can write

$$I_j(t) = \frac{K}{\langle k \rangle} \sum_{n=1}^{N} A_{jn} \int_{-\infty}^{\infty} \int_0^{2\pi} P_q(\theta_n) f_n(\theta_n, \eta_n, t) d\theta_n d\eta_n \tag{11}$$

where we have now evaluated I_j as an average over the ensemble rather than from a single realisation (as in (2)). This is reasonable in the limit of large networks [1].

Multiplying (9) by $\prod_{k \neq j} d\theta_k d\eta_k$ and integrating we obtain

$$\frac{\partial f_j}{\partial t} = -\frac{\partial}{\partial \theta_j} \left\{ \left[1 - \cos\theta_j + (1 + \cos\theta_j)(\eta_j + I_j) \right] f_j \right\} \tag{12}$$

A network of theta neurons is known to be amenable to the use of the Ott/Antonsen ansatz [27, 20, 14] so we write

$$f_j(\theta_j, \eta_j, t) = \frac{g(\eta_j)}{2\pi} \left[1 + \sum_{k=1}^{\infty} \{\alpha_j(\eta_j, t)\}^k e^{ik\theta_j} + \sum_{k=1}^{\infty} \{\bar{\alpha}_j(\eta_j, t)\}^k e^{-ik\theta_j} \right]. \tag{13}$$

The dependence on θ_j is written as a Fourier series where the kth coefficient is the kth power of a function α_j. Substituting this into (12) and (11) we find that α_j satisfies

$$\frac{\partial \alpha_j}{\partial t} = -i \left[\frac{\eta_j + I_j - 1}{2} + (1 + \eta_j + I_j)\alpha_j + \left(\frac{\eta_j + I_j - 1}{2} \right) \alpha_j^2 \right] \tag{14}$$

and

$$I_j(t) = \frac{K}{\langle k \rangle} \sum_{n=1}^{N} A_{jn} \int_{-\infty}^{\infty} H(\alpha_j(\eta_j, t); q) d\eta_n \tag{15}$$

where

$$H(\alpha;q)=a_q\left[C_0+\sum_{n=1}^{q}C_n(\alpha^n+\bar{\alpha}^n)\right] \tag{16}$$

where an overbar indicates complex conjugate, and

$$C_n=\sum_{k=0}^{q}\sum_{m=0}^{k}\frac{\delta_{k-2m,n}q!(-1)^k}{2^k(q-k)!m!(k-m)!} \tag{17}$$

Assuming that g is a Lorentzian:

$$g(\eta)=\frac{\Delta/\pi}{(\eta-\eta_0)^2+\Delta^2} \tag{18}$$

we can use contour integration to evaluate the integral in (15), and evaluating (14) at the appropriate pole of g we obtain

$$\frac{dz_j}{dt}=\frac{-i(z_j-1)^2}{2}+\frac{(z_j+1)^2}{2}\left[-\Delta+i\eta_0+iJ_j\right] \tag{19}$$

where

$$J_j=\frac{K}{\langle k\rangle}\sum_{n=1}^{N}A_{jn}H(z_n;q) \tag{20}$$

and $z_j=\langle e^{i\theta_j}\rangle$, where the expected value is taken over the ensemble.

Now (19) is a set of N coupled complex ODEs, so we have not simplified the original network (1) in the sense of decreasing the number of equations to solve. However, the states of interest are often *fixed points* of (19) (but not of (1)), and can thus be found and followed as parameters are varied. At this point the network we consider, with connectivity given by A, is arbitrary. If A was a circulant matrix, for example, this would represent a network of neurons on a circle, where the strength of coupling between neurons depends only on the distance between them [14].

2.2. **Lumping by degree.** The next step is to assume that for a large enough network, the dynamics of neurons with the same degrees will behave similarly [2]. Such an assumption has been made a number of times in the past [13, 12, 28]. We thus associate with each neuron the degree vector $\boldsymbol{k}=(k^{\text{in}},k^{\text{out}})$ and assume that the value of z for all neurons with a given $\boldsymbol{k}$ are similar. There are $N_{\boldsymbol{k}}=N_{k^{\text{in}}}N_{k^{\text{out}}}$ distinct degrees where $N_{k^{\text{in}}}$ and $N_{k^{\text{out}}}$ are the number of distinct in- and out-degrees, respectively. We define b_s to be the order parameter for neurons with degree $\boldsymbol{k}_s$, where $s\in[1,N_{\boldsymbol{k}}]$, and now derive equations for the evolution of the b_s.

Let $\boldsymbol{z}$ be the vector of ensemble states z_j, where $j\in[1,N]$ and the degree index of neuron j be $d(j)$, such that $\boldsymbol{k}_{d(j)}$ is its degree. We assume that for all neurons with the same degree $\boldsymbol{k}_{d(j)}=\boldsymbol{k}_s$ the ensemble state z_j is similar in sufficiently large networks and thus we only care about the mean value $\langle z_j\rangle_{d(j)=s}=b_s$ with $s\in[1,N_{\boldsymbol{k}}]$. We say that degree $\boldsymbol{k}_s$ occurs h_s times and thus write

$$\boldsymbol{b}=C\boldsymbol{z}, \tag{21}$$

where the $N_{\boldsymbol{k}} \times N$ matrix C has h_s entries in row s, each of value $1/h_s$, at positions j where $d(j) = s$ and zeros elsewhere, i.e. $C_{sj} = \delta_{s,d(j)}/h_s$ with δ being the Kronecker delta.

To find the time derivative of $\boldsymbol{b}$ we need to express $\boldsymbol{z}$ in terms of $\boldsymbol{b}$, which we do with an $N \times N_{\boldsymbol{k}}$ matrix B which assigns to z_j the corresponding b_s value, such that

$$\boldsymbol{z} = B\boldsymbol{b}, \tag{22}$$

with components $B_{js} = \delta_{d(j),s}$. Note that $CB = I_{N_{\boldsymbol{k}}}$, the $N_{\boldsymbol{k}} \times N_{\boldsymbol{k}}$ identity matrix. Differentiating (21) with respect to time, inserting (19) into this and writing $\boldsymbol{z}$ in terms of $\boldsymbol{b}$ using (22) we obtain

$$\begin{aligned} \dot{b_s} = &\underbrace{\sum_{j=1}^{N} C_{sj} \left[-i\frac{\left(\sum_{t=1}^{N_{\boldsymbol{k}}} B_{jt} b_t - 1\right)^2}{2} + i\frac{\left(\sum_{t=1}^{N_{\boldsymbol{k}}} B_{jt} b_t + 1\right)^2}{2} (\eta_0 + i\Delta) \right]}_{\text{local}} \\ &+ \underbrace{\sum_{j=1}^{N} C_{sj} \left[i\frac{\left(\sum_{t=1}^{N_{\boldsymbol{k}}} B_{jt} b_t + 1\right)^2}{2} J_j \right]}_{\text{non-local}}. \end{aligned} \tag{23}$$

Considering that for all t there is only a single non-zero entry B_{jt}, equal to 1, the identity

$$\left(\sum_{t=1}^{N_{\boldsymbol{k}}} \underbrace{B_{jt}}_{=\delta_{d(j),t}} b_t \right)^n = b_{d(j)}^n \tag{24}$$

holds for any power n. Further we find that

$$\sum_{j=1}^{N} \underbrace{C_{sj}}_{=1/h_s \cdot \delta_{s,d(j)}} b_{d(j)} = b_s. \tag{25}$$

Thus, the local term in (23) is

$$\dot{b_s}^{\text{local}} = -i\frac{(b_s - 1)^2}{2} + i\frac{(b_s + 1)^2}{2} (\eta_0 + i\Delta). \tag{26}$$

For the non-local term we write

$$\begin{aligned} \dot{b_s}^{\text{non-local}} &= \sum_{j=1}^{N} \frac{1}{h_s} \delta_{s,d(j)} i \frac{\left(b_{d(j)} + 1\right)^2}{2} J_j \\ &= i\frac{(b_s + 1)^2}{2} \underbrace{\sum_{j=1}^{N} \frac{1}{h_s} \delta_{s,d(j)} J_j}_{=\sum_{j=1}^{N} C_{sj} J_j = \tilde{J}_s}, \end{aligned} \tag{27}$$

where $\tilde{J}_s$ describes the synaptic current of the ensemble equations averaged over nodes sharing the same degree $\boldsymbol{k}_s$. The identity (24) also applies to (20), so that

$$H(z_n;q) = H\left(\sum_{t=1}^{N_{\boldsymbol{k}}} B_{nt} b_t; q\right) = \sum_{t=1}^{N_{\boldsymbol{k}}} B_{nt} H(b_t;q) \tag{28}$$

and the current can be written as

$$\begin{aligned} \tilde{J}_s &= \sum_{j=1}^{N} C_{sj} \frac{K}{\langle k \rangle} \sum_{n=1}^{N} A_{jn} \sum_{t=1}^{N_{\boldsymbol{k}}} B_{nt} H(b_t;q) \\ &= \frac{K}{\langle k \rangle} \sum_{t=1}^{N_{\boldsymbol{k}}} \underbrace{\sum_{j=1}^{N} \sum_{n=1}^{N} C_{sj} A_{jn} B_{nt}}_{E_{st}} H(b_t;q) \end{aligned} \tag{29}$$

The effective connectivity between neurons with different degrees is therefore expressed in the matrix $E = CAB$ and we end up with equations governing the b_s:

$$\frac{db_s}{dt} = \frac{-i(b_s-1)^2}{2} + \frac{(b_s+1)^2}{2}\left[-\Delta + i\eta_0 + i\tilde{J}_s\right] \tag{30}$$

where

$$\tilde{J}_s = \frac{K}{\langle k \rangle} \sum_{t=1}^{N_{\boldsymbol{k}}} E_{st} H(b_t;q) \tag{31}$$

These equations are of the same form as (19)-(20) except that A has been replaced by E. Note that the connectivity matrix A is completely general; we have only assumed that neurons with the same degrees behave in the same way. We are not aware of a derivation of this form being previously presented.

3. Network assembly

We are interested in the effects of degree assortativity on the dynamics of the network of neurons. We will choose a default network with no assortativity and then introduce one of the four types of assortativity and investigate the changes in the network's dynamics. Our default network is of size $N = 5000$ neurons where in- and out-degrees k for each neuron are independently drawn from the interval $[750, 2000]$ with probability $P(k) \sim k^{-3}$. We create networks using the configuration model [25], then modify them using algorithms which introduce assortativity and then remove multiple connections between nodes (or multi-edges) (described in Appendix A). Further, to observe any influence of multi-edges on the dynamics investigated here, we also developed a novel network assembly technique permitting introduction of very high densities of multi-edges also described in the Appendix; we refer to this novel technique as the "permutation" method. We choose as our default parameters $\eta_0 = -2, \Delta = 0.1, K = 3$, for which a default network approaches a stable fixed point. The sharpness of the synaptic pulse function is set to $q = 2$ for all simulations.

We first check the validity of averaging over an infinite ensemble. We assemble 20 different default networks and for each, run (19)-(20) to a steady state and calculate the order parameter z, the mean of $B\boldsymbol{b}$. The real part of z is plotted in orange in Fig. 2. For

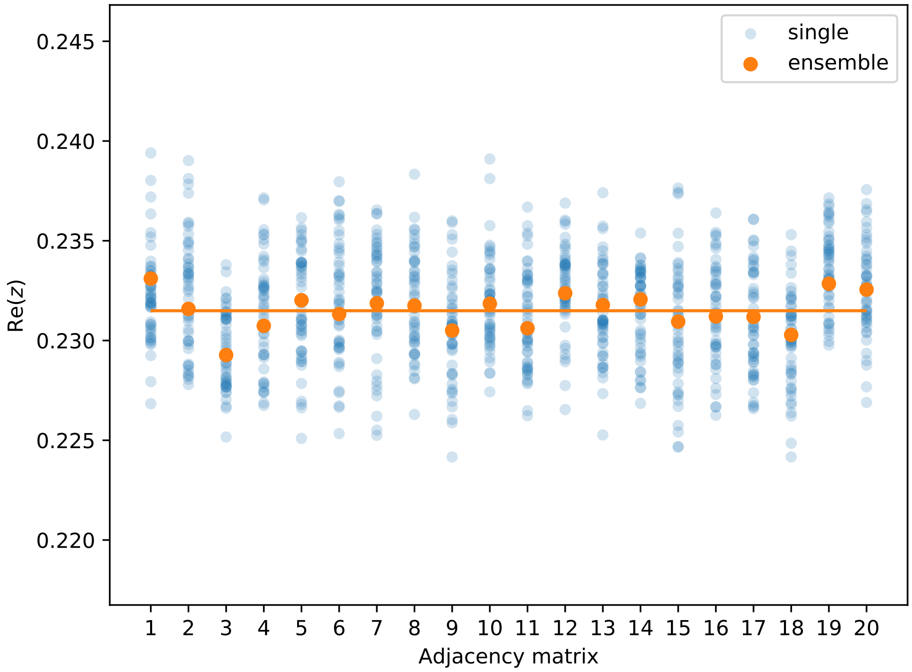

FIGURE 2. Orange circles: steady state of (19)-(20) for 20 different default networks. Blue circles: results from 50 different realisations of the η_i for (1)-(2), for each network. Parameters: $\eta_0 = -2, \Delta = 0.1, K = 3$. The orange line marks the ensemble mean value.

each of these networks we then generated 50 realisations of the η_i's and ran (1)-(2) for long enough that transients had decayed, and then measured the corresponding order parameter for the network of individual neurons

$$R = \frac{1}{N}\sum_{j=1}^{N} e^{i\theta_j} \tag{32}$$

and plotted its real part in blue in Fig. 2. Note that the orange circles always lie well within the range of values shown in blue. The fact that deviations within the 50 realisations are small relative to the value obtained by averaging over an infinite ensemble provide evidence for the validity of this approach, at least for these parameter values.

We also investigate the influence of multi-connections (i.e. more than one connection) between neurons on the network dynamics. The configuration model creates a network in which the neuron degrees are exactly those specified by the choice from the appropriate distribution, but typically results in both self-connections and multiple connections between neurons. We have an algorithm for systematically removing such connections while preserving the degrees, and found that removing such edges has no significant effect (results not shown). We also have an algorithm (see "Permutation Method" in Appendix A) for *increasing* the number of multi-edges from the number found using the default configuration model. This novel network assembly method meets specified neuron degrees and also produces specified densities of multi-connections ranging from none to 97%; see Fig. 3 for the results of such calculations. We see that only when the

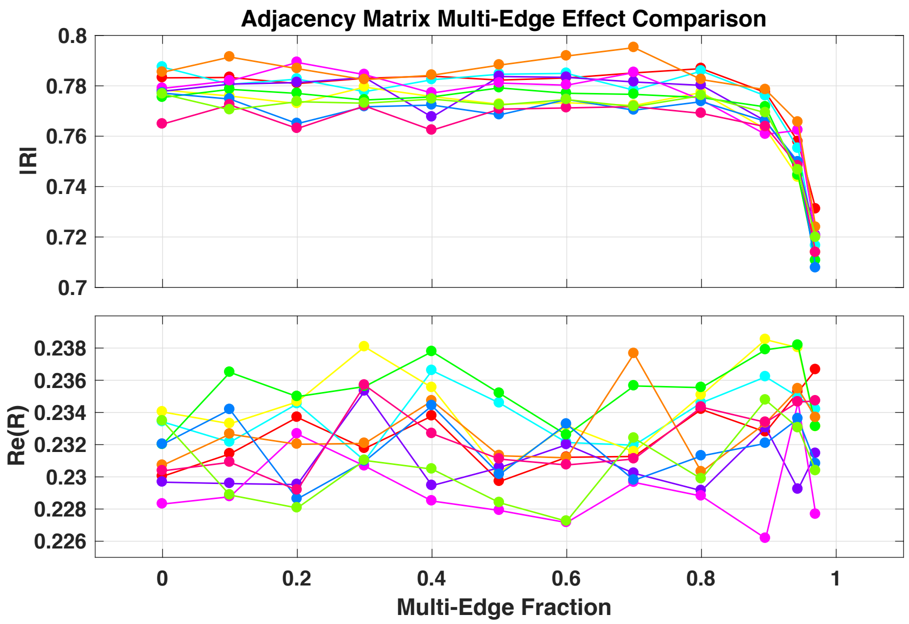

FIGURE 3. Comparison of steady-state values of the order parameter (top: magnitude of R; bottom: Re(R)) over a suite of adjacency matrices with varied densities of multi-edge connections ranging from none to 97%. Higher densities of multi-edges were obtained, but assortativities exceeded the target neutral values of ± 0.005. Values shown are from simulations of (1)-(2) after initial transients decay (i.e., time t ≥ 40). Each of these 10 curves correspond to a unique realisation of default ηs from the distribution $g(\eta)$. Parameters: $N = 5000, \eta_0 = -2, \Delta = 0.1, K = 3, q = 2$.

fraction of multi-edges approaches 90% do we see a significant effect. However, in our simulations we use simple graphs without multi-edges.

3.1. **Assortativity.** For a given matrix A we can measure its assortativity by calculating the four Pearson correlation coefficients $r(\alpha, \beta)$ with $\alpha, \beta \in [\text{in}, \text{out}]$ which read

$$r(\alpha,\beta) = \frac{\sum_{e=1}^{N_e}({}^s k_e^\alpha - \langle {}^s k^\alpha \rangle)({}^r k_e^\beta - \langle {}^r k^\beta \rangle)}{\sqrt{\sum_{e=1}^{N_e}({}^s k_e^\alpha - \langle {}^s k^\alpha \rangle)^2}\sqrt{\sum_{e=1}^{N_e}({}^r k_e^\beta - \langle {}^r k^\beta \rangle)^2}} \tag{33}$$

where

$$\langle {}^s k^\alpha \rangle = \frac{1}{N_e}\sum_{e=1}^{N_e} {}^s k_e^\alpha \qquad \text{and} \qquad \langle {}^r k^\beta \rangle = \frac{1}{N_e}\sum_{e=1}^{N_e} {}^r k_e^\beta, \tag{34}$$

N_e being the number of connections and the leading superscript s or r refers to the sending or receiving neuron of the respective edge. For example the sending node's in-degree of the second edge would be ${}^s k_2^{in}$. Note that there are four mean values to compute.

We introduce assortativity by randomly choosing two edges and swapping postsynaptic neurons when doing so would increase the target assortativity coefficient [30]. An edge (i, j) is directed from neuron j to neuron i. In order to know whether the pair

(i,j) and (h,l) should be rewired or left untouched, we compare their contribution to the covariance in the numerator of (33):

$$c_{\parallel} = c((i,j),(h,l))$$
$$= \left(k_j^{\alpha} - \langle {}^s k^{\alpha}\rangle\right)\left(k_i^{\beta} - \left\langle {}^r k^{\beta}\right\rangle\right) + \left(k_l^{\alpha} - \langle {}^s k^{\alpha}\rangle\right)\left(k_h^{\beta} - \left\langle {}^r k^{\beta}\right\rangle\right); \tag{35}$$
$$c_{\chi} = c((i,l),(h,j))$$
$$= \left(k_l^{\alpha} - \langle {}^s k^{\alpha}\rangle\right)\left(k_i^{\beta} - \left\langle {}^r k^{\beta}\right\rangle\right) + \left(k_j^{\alpha} - \langle {}^s k^{\alpha}\rangle\right)\left(k_h^{\beta} - \left\langle {}^r k^{\beta}\right\rangle\right). \tag{36}$$

If $c_{\chi} > c_{\parallel}$ we replace the edges (i,j) and (h,l) by (i,l) and (h,j), respectively, otherwise we do not, and continue by randomly choosing another pair of edges. Algorithm 1 (see Appendix A) demonstrates a scheme for reaching a certain target assortativity coefficient.

We investigate the effects of different types of assortativity (see Fig 1) in isolation. We thus need a family of networks parametrised by the relevant assortativity coefficient. Algorithm 1 is used to create a network with a specific value of one of the assortativity coefficients, but especially for high values of assortativity it may be that in doing so a small amount of assortativity of a type other than the intended one is introduced. Accordingly, it may be necessary to examine all types of assortativity and apply the mixing scheme to reduce other types back to zero, and then (if necessary) push the relevant value of assorativity back to its target value. We do multiple iterations of these mixing rounds until all assortativities are at their target values (which may be 0) within a range of ± 0.005. We use Algorithm 1 with a range of target assortativities r, and for each value, store the connectivity matrix A and thus form the parametrised family $E(r)$. We do this for the four types of assortativity.

We have chosen to use the configuration model to create networks with given degree sequences and then introduced assortativity by swapping edges. Although we developed our novel "permutation" method as well (see Appendix A), that method was designed for assembling adjacency matrices with desired multi-edge densities and was applied only for that aspect. By contrast, another common adjacency network assembly technique, that of Chung and Lu [4] together with an analytical expression for assortativity (as in [2]), proved inadequate. We found that the latter approach significantly alters the degree distribution for large assortativity, whereas the configuration model combined with our mixing algorithm does not change degrees at all. For our default network this approach allows us to introduce assortativity of any one kind up to $r = \pm 0.5$.

4. Implementation

For networks of the size we investigate it is impractical to consider each distinct in- and out-degree (because E will be very large and sparse). Due to the smoothness of the degree dependency of $b(\boldsymbol{k})$ we coarse-grain in degree space by introducing "degree clusters" — lumping all nodes with a range of degrees into a group with dynamics described by a single variable. Let there be $N_{c^{\text{in}}}$ clusters in in-degree and $N_{c^{\text{out}}}$ clusters in out-degree, with a total of $N_{\boldsymbol{c}} = N_{c^{\text{in}}} \cdot N_{c^{\text{out}}}$ degree clusters. The matrix C then is an $N_{\boldsymbol{c}} \times N$ matrix and constructed as previously, except that $d(j)$ is not the degree index of neuron j, but the cluster index and s is the cluster index running from 1 to

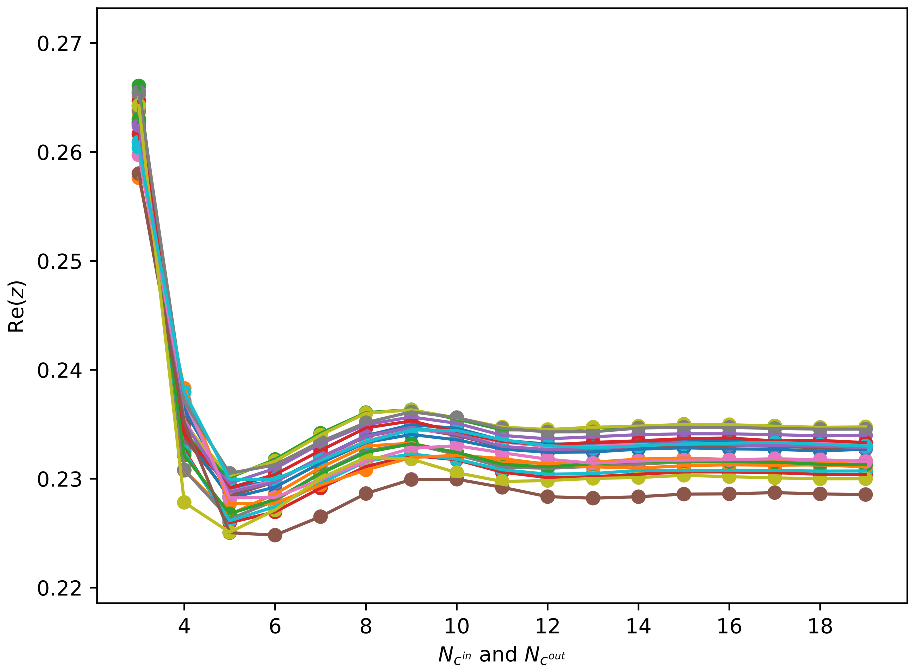

FIGURE 4. Real part of z at steady state for 20 different default adjacency matrices (indicated by different colors), as the number of clusters in degree space is varied. Parameters: $\eta_0 = -2, \Delta = 0.1, K = 3$.

$N_{\boldsymbol{c}}$. Similarly for the matrix B. There are multiple options for how to combine degrees into a cluster. The cluster index of a neuron can be computed linearly, corresponding to clusters of equal size in degree space. However, with this approach, depending on the degree distribution, some of the clusters may be empty or hardly filled, resulting in poor statistics. To overcome this issue, the cumulative sum of in- and out-degree distribution can be used to map degrees to cluster indices. Thus, clusters are more evenly filled and at the same time regions of degree space with high degree probability are more finely sampled. The dynamical equations (30)-(31) are equally valid for describing degree cluster dynamics with $s, t \in [1, N_{\boldsymbol{c}}]$ and $E = CAB$, where C and B are cluster versions of their previous definitions.

To check the effect of varying the number of clusters we generate 20 default matrices and then generate the corresponding matrix E with varying numbers of clusters ($N_{c^{\text{in}}}$ and $N_{c^{\text{out}}}$ are equal), then run (30)-(31) to a steady state and plot the real part of z in Fig. 4. We see that the order parameter is well approximated using as little as about $N_{c^{\text{in}}} = N_{c^{\text{out}}} = 10$ degree clusters. Beyond that, fluctuations between different network realisations exceed the error introduced by clustering. In our simulations we stick to the choice of 10 degree clusters per in- and out-degree space.

Having performed this clustering, we find that it is possible to represent E using a low-rank approximation, calculated using singular value decomposition. Thus for a fixed r we have

$$E = USV^T \tag{37}$$

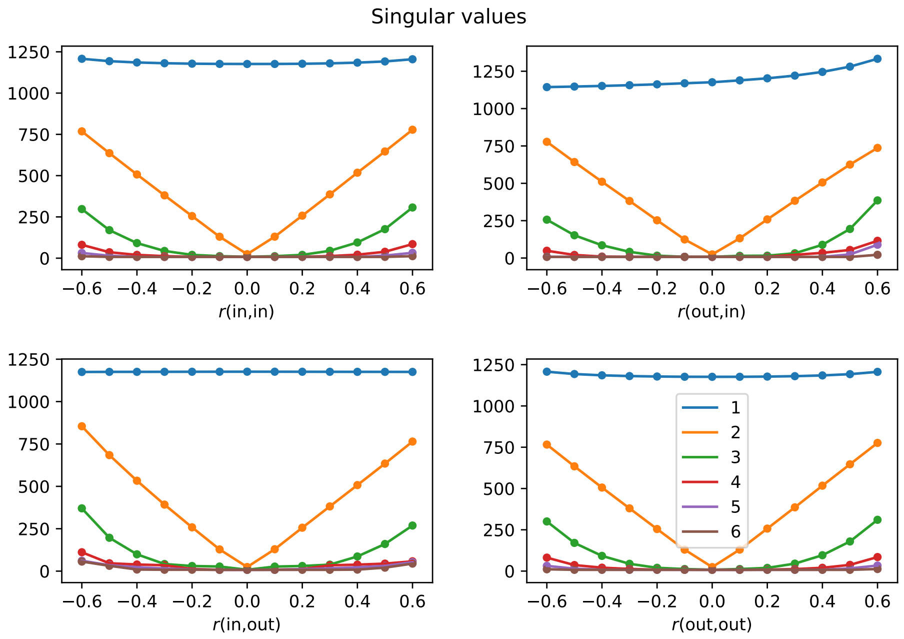

FIGURE 5. Six largest singular values of the SVD decomposition of E as a function of assortativity coefficent, for 4 types of assortativity.

where S is a diagonal matrix with decreasing entries, called singular values, and U and V are unitary matrices. In Fig. 5 we plot the largest 6 singular values of E as function the assortativity coefficient, for the 4 types of assortativity. Even for large $|r|$ the singular values decay very quickly, thus a low-rank approximation is possible. We choose a rank-3 approximation, so approximate E by

$$E(r) \approx \left[\begin{array}{c|c|c} u_1(r) & u_2(r) & u_3(r) \end{array} \right] \begin{bmatrix} s_1(r) & 0 & 0 \\ 0 & s_2(r) & 0 \\ 0 & 0 & s_3(r) \end{bmatrix} \begin{bmatrix} v_1^T(r) \\ \hline v_2^T(r) \\ \hline v_3^T(r) \end{bmatrix} \tag{38}$$

where u_i is the ith column of U, similarly for v_i and V, and s_i is the ith singular value. We have such a decomposition at discrete values of r and use cubic interpolation to evaluate $E(r)$ for any r. This decomposition means that the multiplication in (31) can be evaluated quickly using 3 columns of U and V rather than the full $N_{\boldsymbol{c}} \times N_{\boldsymbol{c}}$ matrix E.

We note that the components for the approximation of $E(r)$ are calculated once and then stored, making it very easy to systematically investigate the effects of varying any of the parameters η_0, Δ, K and q (governing the sharpness of the pulse function (3)).

5. Results

5.1. **Excitatory coupling.** We take $K = 3$ to model a network with only excitatory connections. To study the dynamical effect of assortativity we generate positive and negative ($r = \pm 0.2$) assortative networks of the four possible kinds and follow fixed

points of (30)-(31) as a function of η_0, and compare results with a neutral ($r = 0$) network. We use pseudo-arc-length continuation [15, 11].

To calculate the mean frequency over the network we evaluate $\boldsymbol{z} = B\boldsymbol{b}$ and then use the result that if the order parameter at a node is z, then the frequency of neurons at that node is [16, 23]

$$\frac{1}{\pi}\text{Re}\left(\frac{1-\bar{z}}{1+\bar{z}}\right) \tag{39}$$

Averaging these gives the mean frequency.

Results are shown in Figure 6, where we see quite similar behaviour in each case: apart from a bistable region containing two stable and one unstable fixed point, there is only a single stable fixed point present. Further, the two assortativity types (out,in) and (out,out) apparently do not affect the dynamics, whereas the saddle-node bifurcations marking the edges of the bistable region move slightly for (in,out) and significantly for (in,in) assortativity. Following the saddle-node bifurcations for the latter two cases we find the results shown in Figure 7. We have performed similar calculations for different networks with the same values of assortativity and found similar results.

5.2. **Inhibitory coupling.** We choose $K = -3$ to model a network with only inhibitory coupling. Again, we numerically continue fixed points for zero, positive and negative assortativity ($r = 0, \pm 0.2$) as η_0 is varied and obtain the curves shown in Figure 8. Consider the lower left plot. For large η_0 the system has a single stable fixed point which undergoes a supercritical Hopf bifurcation as η_0 is decreased, creating a stable periodic orbit. This periodic orbit is destroyed in a saddle-node bifurcation on an invariant circle (SNIC) bifurcation at lower η_0, forcing the oscillations to stop. Decreasing η_0 further, two unstable fixed points are destroyed in a saddle-node bifurcation. In contrast with the case of excitatory coupling, oscillations in the average firing rate are seen. These can be thought of as partial synchrony, since some fraction of neurons in the network have the same period and fire at similar times to cause this behaviour. The period of this macroscopic oscillation tends to infinity as the SNIC bifurcation is approached, as shown in the inset of the lower left panel in Fig. 8.

As in the excitatory case, we see that assortativities of type (out,in) and (out,out) have no influence on the dynamics in this scenario. However, type (in,out) does have a small effect, slightly moving bifurcation points (top right panel in Fig. 8). Type (in,in) has the strongest effect, resulting in a qualitative change in the bifurcation scenario for large enough assortativity: there is a region of bistability between either two fixed points or a fixed point and a periodic orbit. This is best understood by following the bifurcations in the top panels of Fig. 8 as r is varied, as shown in Figure 9. There is one fixed point in regions A, B and D, and three in region C. For (in,out) assortativity there is a stable periodic orbit in region B and never any bistability.

We now describe the case for (in,in) assortativity. For negative and zero r the scenario is the same as for the other three types, but as r is increased there is a Takens-Bogdanov bifurcation where regions C,D,E and F meet, leading to the creation of a curve of homoclinic bifurcations, which is destroyed at another codimension-two point where there is a homoclinic connection to a non-hyperbolic fixed point [3]. There are stable oscillations

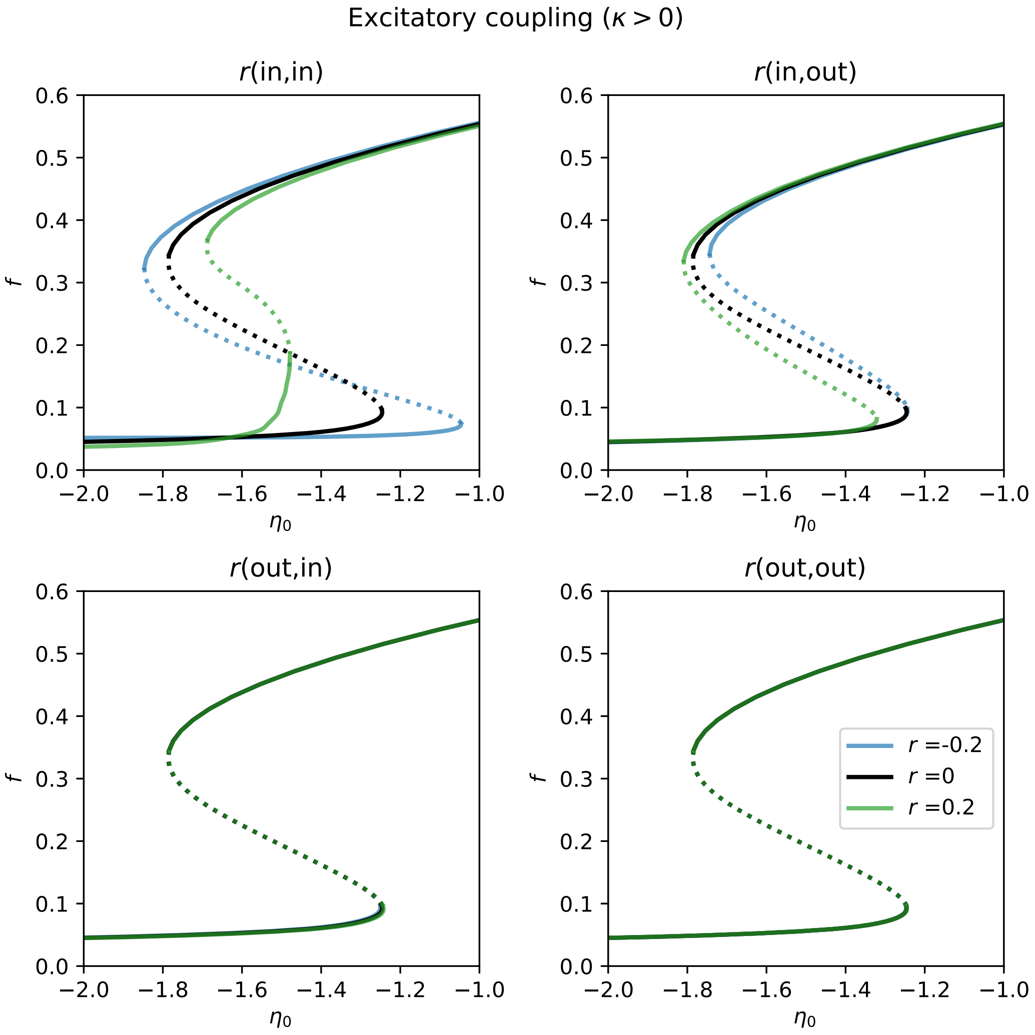

Figure 6. Average firing rate at fixed points of (30)-(31) as a function of η_0, for the 4 types of assortativity. For each type of assortativity curves are plotted for $r = 0$ (black), $r = -0.2$ (blue) and $r = 0.2$ (green). Solid lines indicate stable and dashed lines unstable fixed points. Parameters: $K = 3, \Delta = 0.1$.

in region E, created or destroyed in supercritical Hopf or homoclinic bifurcations. In region F there is bistability between two fixed points.

6. Discussion

We investigated the effects of degree assortativity on the dynamics of a network of theta neurons. We used the Ott/Antonsen ansatz to derive evolution equations for an order parameter associated with each neuron, and then coarse-grained by degree and then degree cluster, obtaining a relatively small number of coupled ODEs, whose dynamics as parameters varied could be investigated using numerical continuation. We found that degree assortativity involving the out-degree of the sending neuron, i.e. (out,in) and (out,out), has no effect on the networks' dynamics. Further, (in,out) assortativity moves bifurcations slightly, but does not lead to substantial differences in dynamical

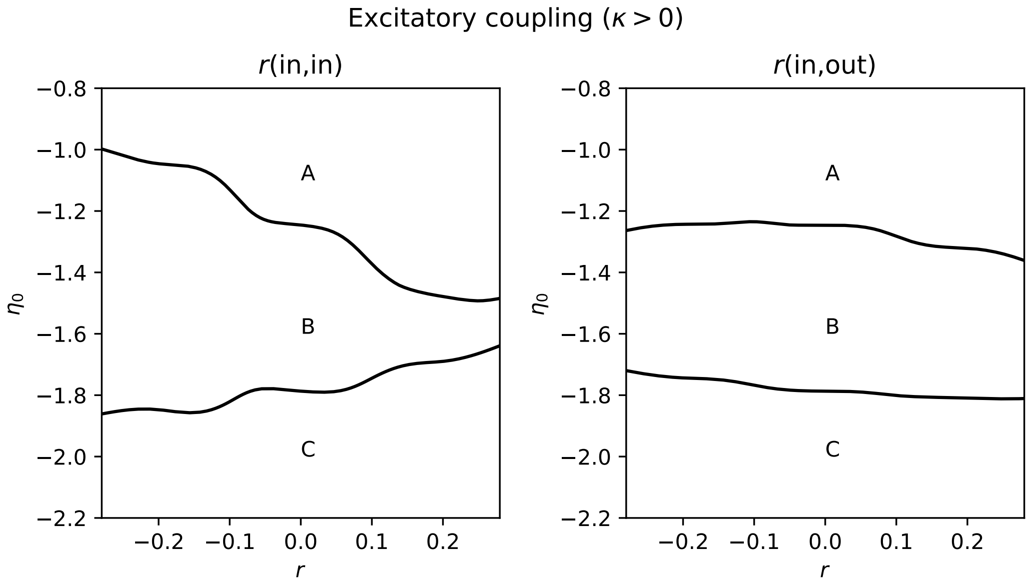

Figure 7. Continuation of the saddle-node bifurcations seen in the upper two panels of Fig. 6 as r is varied. Curves in Figure 6 correspond to vertical slices at $r = 0, \pm 0.2$. The network is bistable in region B and has a single stable fixed point in regions A and C.

behaviour. The most significant effects were caused by creating correlation between in-degrees of the sending and receiving neurons. For our excitatorially coupled example, positive (in,in) assortativity narrows the bistable region, whereas negative assortativity widens it (see Fig. 7). In the inhibitory case introducing negative assortativity increased the amplitude of network oscillations and extended their range to slightly larger η_0. On the contrary, positive (in,in) assortativity in this network has an opposite effect and eventually stops oscillations (see Fig. 9).

The most similar work to ours is that of [2]. These authors also considered a network of the form (1)-(2) and by assuming that the dynamics depend on only a neuron's degree and that the η_j are chosen from a Lorentzian, and using the Ott/Antonsen ansatz, they derived equations similar to (30)-(31). The difference in formulations is that rather than a sum over entries of E (in (31)), [2] wrote the sum as

$$\sum_{\mathbf{k}'} P(\mathbf{k}')a(\mathbf{k}' \to \mathbf{k}) \tag{40}$$

where $P(\mathbf{k})$ is the degree distribution and $a(\mathbf{k}' \to \mathbf{k})$ is the assortativity function, which specifies the probability of a link from a node with degree $\mathbf{k}'$ to one with degree $\mathbf{k}$ (given that such neurons exist). They then chose a particular functional form for a and briefly presented the results of varying one type of assortativity (between k'_{in} and k_{out}). In contrast, our approach is far more general (since *any* connectivity matrix A can be reduced to the corresponding E, the only assumption being that the dynamics are determined by a neuron's degree). We also show the results of a wider investigation into the effects of assortativity.

This alternative presentation also explains why E can be well approximated with a low-rank approximation. If the in- and out-degrees of a single neuron are independent,

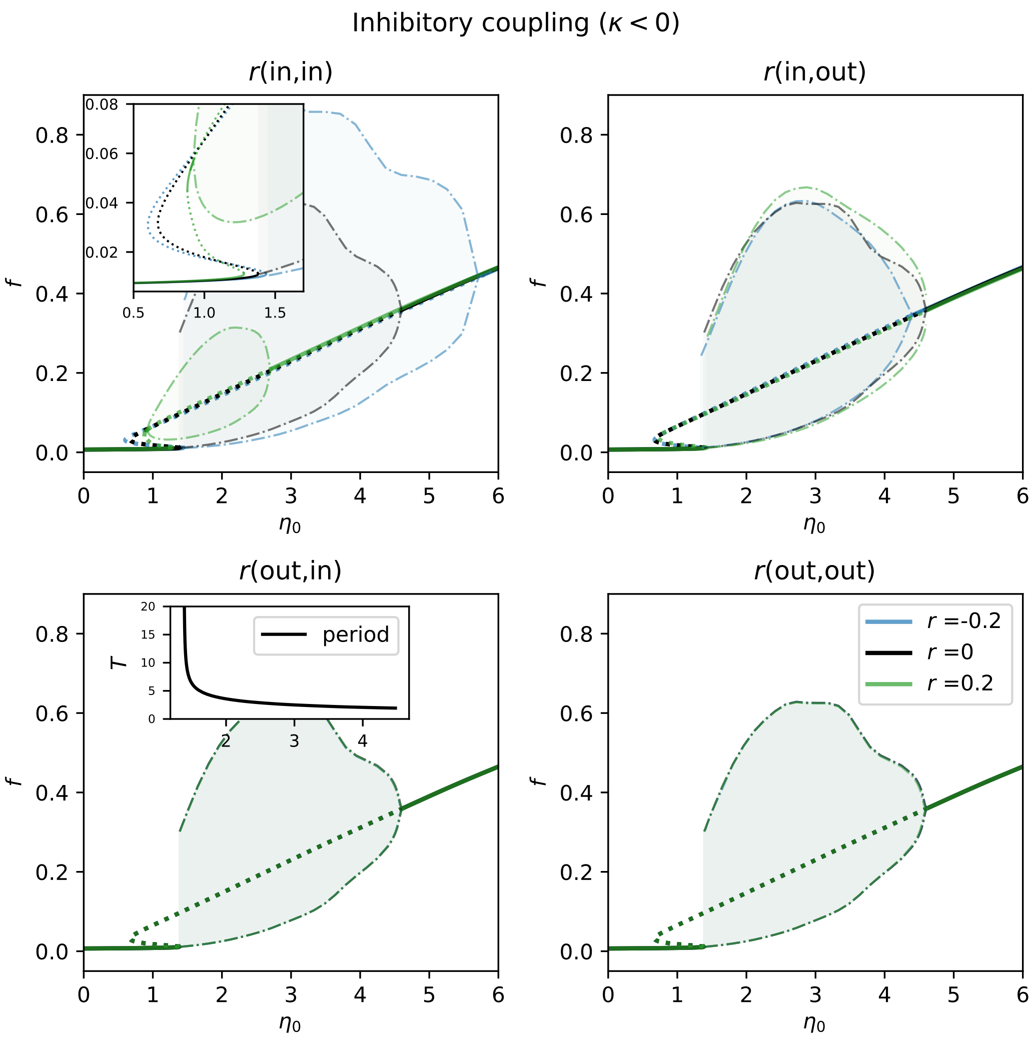

FIGURE 8. Average firing rate at fixed points of (30)-(31) as a function of η_0, for the 4 types of assortativity. For each type of assortativity curves are plotted for $r = 0$ (black), $r = -0.2$ (blue) and $r = 0.2$ (green). In addition there are oscillations in certain regions and dash-dotted lines outline the minimal and maximal firing rate over one period of oscillation. The (in,in)-plot in the top left corner contains a zoom of rest of the panel, and the (out,in)-plot contains a subplot with the oscillation's period for $r = 0$ and which is aligned with the outer η_0 axis.

$P(\mathbf{k}') = P_i(k'_{in})P_o(k'_{out})$, and with neutral assortativity, $a(\mathbf{k}' \to \mathbf{k}) = k'_{out}k_{in}/(N\langle k\rangle)$. Thus

(41)
$$\sum_{\mathbf{k}'} P(\mathbf{k}')a(\mathbf{k}' \to \mathbf{k})H(b(\mathbf{k}'); q) = \frac{k_{in}}{N\langle k\rangle} \sum_{k'_{out}} \sum_{k'_{in}} k'_{out} P_i(k'_{in}) P_o(k'_{out}) H(b(k'_{out}, k'_{in}); q)$$

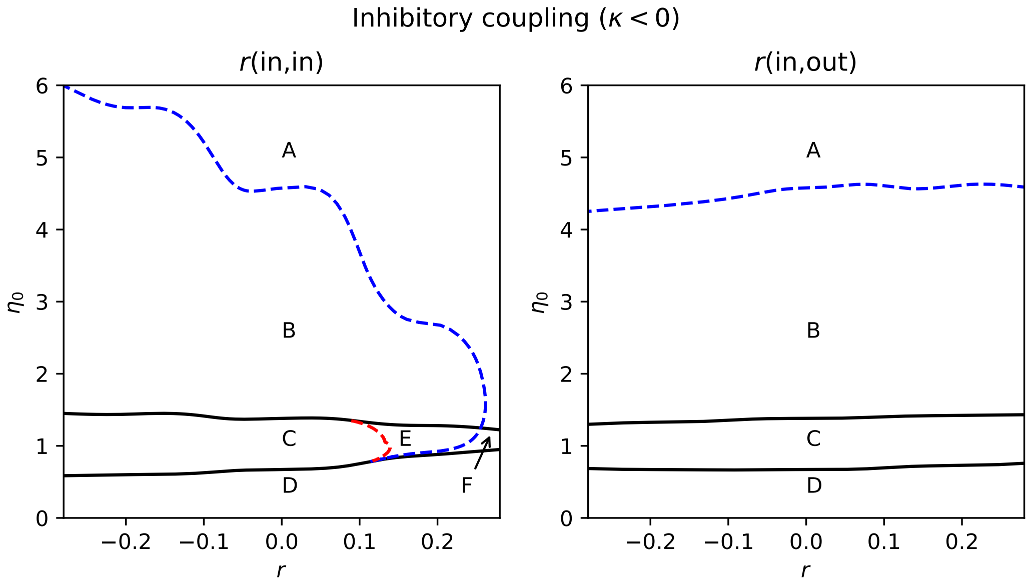

FIGURE 9. Continuation of bifurcations seen in upper panels of Fig. 8. Solid black lines indicate saddle-node bifurcations, dashed blue is a Hopf bifurcation and dashed red a homoclinic bifurcation. Curves in Figure 8 can be understood as vertical slices through the respective plot at $r = 0, \pm 0.2$. See text for explanation of labels.

This term contributes to the input current to a neuron with degree $\mathbf{k} = (k_{in}, k_{out})$, but is independent of k_{out}. Thus the state of a neuron depend only on its in-degree, so

$$\sum_{\mathbf{k}'} P(\mathbf{k}')a(\mathbf{k}' \to \mathbf{k})H(b(\mathbf{k}'); q) = \frac{k_{in}}{N} \sum_{k'_{in}} P_i(k'_{in})H(b(k'_{in}); q) \tag{42}$$

Comparing with (31) we see that $E = \mathbf{c}^T\mathbf{d}$ where $\mathbf{c} = (k_{in}^1, k_{in}^2 \dots k_{in}^{N_{k^{in}}})/N$ and $\mathbf{d} = (P_i(k_{in}^1), P_i(k_{in}^2) \dots, P_i(k_{in}^{N_{k^{in}}}))$, i.e. E is a rank-one matrix. Varying assortativity within the network is then a perturbation away from this, with the effects appearing in the second (and third) singular values in the SVD decomposition of E.

A limitation of our study is that we considered only networks of fixed size with the same distributions of in- and out-degrees, and a specific distribution of these degrees. However, our approach does not rely on this and could easily be adapted to consider other types of networks, although we expect it to become less valid as both the average degree and number of neurons in the network decrease. We have also only considered theta neurons, but since a theta neuron is the normal form of a Type I neuron, we expect similar networks of other Type I neurons to behave similarly to the networks considered here. The approach presented here could also be used to efficiently investigate the effects of correlated heterogeneity, where either the mean or width of the distribution of the η_j is correlated with a neuron's in- or out-degree [33, 34, 5]. We could also consider assortativity based on a neuron's intrinsic drive (η_j) [32] rather than its degrees, or correlations between an individual neuron's in- and out-degree [35, 17, 21, 36, 26]. We are currently investigating such ideas.

Acknowledgements: This work is partially supported by the Marsden Fund Council from Government funding, managed by Royal Society Te Apārangi.

Appendix A. Algorithms

We present here the algorithms developed and utlised for adjacency matrix assembly and modification. These include modifications to resulting adjacency matrices produced by the familiar configuration method, and our novel matrix assembly technique we christen the "permutation" method.

A.1. **Assortativity.** Algorithm 1 is used to create a network with a specified degree distribution and values of the four different assortativity coefficients.

A.2. **Permutation Method.** The well-known configuration model for generating adjacency matrices [25] typically includes auto-connections and multiple edge connections with no control over their appearance or proportion, often forcing post-processing removal if none are desired. We developed a novel adjacency matrix assembly technique that, given predefined sequences of in- and out-degrees, permits designating not only whether multiple edges appear but also their proportion — with no post-processing required. Additionally, auto-connections can be included or omitted without manipulating the resulting A. These A's exhibit generally neutral assortativities over all types with exceptions emerging for the highest multi-edge densities we assembled: e.g., 98-99% multi-edges exceed our target neutral assortativity range of ± 0.005, so for purposes of this study these were discarded.

This permutation method is a two-phase approach requiring only two sequences of in- and out-degrees, or k_{in} and k_{out}, respectively, and a target multiple edge connection density, ρ_m^+. We describe this technique briefly here, and with more detail in a subsequent companion publication. These phases are as follows:

(1) Generate an initial matrix, designated $A^{(0)}$, with each node's inbound edge counts (row sums) satisfying k_{in}, yet ignoring k_{out}. Each row of $A^{(0)}$ is filled with nonzero entries comprised of solo- and multiple-edge connections whose sum is k_{in} for each node. Remaining entries along each row are simply filled with zeros out to the Nth column. This resulting $A^{(0)}$ thus adheres to the designated k_{in} and ρ_m^+, but violates k_{out}: all the column sums are incorrect (see Fig. (10)).
(2) Randomly permute each row of $A^{(0)}$, distributing the non-zero entries of solo- and multiple-edge connections into a first sequence of permuted matrices, $A^{(1)}$. Calculate an error distance for $A^{(1)}$ from the designated k_{out} via $e_{out}^{(1)} = k_{out} - k_{out}^{(1)}$. Each entry in $e_{out}^{(1)}$ is used to classify nodes: too many out-bound edges ("donor" nodes), those with too few ("recipient" nodes) and those at their target out-degree ("inert" nodes). We then loop over all the donor nodes, randomly pick a recipient node and exchange edges from the donor to the recipient — if suitable. After each exchange, we update the current permuted matrix, $A^{(i)}$, its corresponding error distance, $e_{out}^{(i)}$, and repeat the process until this error is zero. The final matrix, $A^{(n)}$, after n updates, then satisfies all the designated characteristics if we performed suitable edge exchanges along the way.

Algorithm 1: Assortative mixing.

Randomly pair up all N_e edges of the network with adjacency matrix A and reconnect them at once where preferable with respect to target assortativity r_{target}. Repeat the process until the assortativity coefficient lies within the tolerance. Once overshooting the target coefficient, interpolate the length of a shortened list of edge pairs and reconnect those.

/* compute difference in assortativity */
$\Delta r = r_{\text{target}} - r(A)$;
while $|\Delta r| > tolerance$ **do**
  pair up all edges $[(i, j), (k, l)]$;
  /* compute whether each pair should be reconnected */
  $s_{\Delta r}$ = [true: if reconnection will minimise Δr; false: otherwise];
  /* trial reconnection */
  $A^* = \text{copy}(A)$;
  reconnect edges in A^* according to $s_{\Delta r}$;
  $\Delta r^* = r_{\text{target}} - r(A^*)$;
  if $\text{sign}(\Delta r^*) \neq \text{sign}(\Delta r)$ **then**
    /* r(A*) is already beyond the target: */
    /* limit number of edges for reconnection process */
    interpolation data Γ: $(0, r(A)), (N_e/2, r(A^*))$;
    while $|\Delta r^*| > tolerance$ **do**
      interpolate (L, r_{target}) using Γ;
      s_{limit} = [true: list index $< L$; false: list index $> L$];
      /* trial selection and reconnection */
      $s^* = s_{\Delta r} \wedge s_{\text{limit}}$;
      $A^* = \text{copy}(A)$;
      reconnect edges in A^* according to s^*;
      add $(L, r(A^*))$ to Γ;
      $\Delta r^* = r_{\text{target}} - r(A^*)$;
    end
  end
  $A = A^*$;
  $\Delta r = \Delta r^*$;
end

References

[1] Gilad Barlev, Thomas M Antonsen, and Edward Ott. The dynamics of network coupled phase oscillators: An ensemble approach. *Chaos*, 21(2):025103, 2011.

[2] Sarthak Chandra, David Hathcock, Kimberly Crain, Thomas M. Antonsen, Michelle Girvan, and Edward Ott. Modeling the network dynamics of pulse-coupled neurons. *Chaos*, 27(3):033102, 2017.

[3] Shui-Nee Chow and Xiao-Biao Lin. Bifurcation of a homoclinic orbit with a saddle-node equilibrium. *Differential and Integral Equations*, 3:435–466, 01 1990.

$$\mathbf{A}^{(0)} = \begin{array}{c} \\ n_{in}^1 \\ n_{in}^2 \\ n_{in}^3 \\ n_{in}^4 \\ n_{in}^5 \\ n_{in}^6 \\ n_{in}^7 \\ n_{in}^8 \\ n_{in}^9 \end{array} \begin{array}{ccccccccc|c} n_{out}^1 & n_{out}^2 & n_{out}^3 & n_{out}^4 & n_{out}^5 & n_{out}^6 & n_{out}^7 & n_{out}^8 & n_{out}^9 & \mathbf{k_{in}} \\ 1 & 1 & 0 & 0 & 0 & 0 & 0 & 0 & 0 & 2 \\ 1 & 1 & 0 & 0 & 0 & 0 & 0 & 0 & 0 & 2 \\ 1 & 1 & 1 & 0 & 0 & 0 & 0 & 0 & 0 & 3 \\ 1 & 1 & 1 & 0 & 0 & 0 & 0 & 0 & 0 & 3 \\ 1 & 1 & 1 & 1 & 1 & 0 & 0 & 0 & 0 & 5 \\ 1 & 1 & 0 & 0 & 0 & 0 & 0 & 0 & 0 & 2 \\ 1 & 1 & 0 & 0 & 0 & 0 & 0 & 0 & 0 & 2 \\ 1 & 1 & 0 & 0 & 0 & 0 & 0 & 0 & 0 & 2 \\ 1 & 1 & 0 & 0 & 0 & 0 & 0 & 0 & 0 & 2 \end{array}$$

$$\mathbf{A}^{(1)} = \begin{array}{c} \\ n_{in}^1 \\ n_{in}^2 \\ n_{in}^3 \\ n_{in}^4 \\ n_{in}^5 \\ n_{in}^6 \\ n_{in}^7 \\ n_{in}^8 \\ n_{in}^9 \\ \hline \mathbf{k_{out}^1} \end{array} \begin{array}{ccccccccc|c} n_{out}^1 & n_{out}^2 & n_{out}^3 & n_{out}^4 & n_{out}^5 & n_{out}^6 & n_{out}^7 & n_{out}^8 & n_{out}^9 & \mathbf{k_{in}} \\ 0 & 0 & 0 & 0 & 0 & 1 & 1 & 0 & 0 & 2 \\ 0 & 0 & 0 & 0 & 0 & 1 & 1 & 0 & 0 & 2 \\ 0 & 1 & 0 & 1 & 0 & 1 & 0 & 0 & 0 & 3 \\ 0 & 0 & 0 & 0 & 1 & 0 & 1 & 1 & 0 & 3 \\ 0 & 1 & 1 & 1 & 0 & 1 & 1 & 0 & 0 & 5 \\ 0 & 1 & 1 & 0 & 0 & 0 & 0 & 0 & 0 & 2 \\ 0 & 0 & 0 & 0 & 0 & 0 & 1 & 1 & 0 & 2 \\ 0 & 0 & 1 & 0 & 0 & 0 & 0 & 1 & 0 & 2 \\ 0 & 0 & 0 & 0 & 0 & 0 & 1 & 1 & 0 & 2 \\ \hline 0 & 3 & 3 & 2 & 1 & 4 & 6 & 4 & 0 & \end{array}$$

FIGURE 10. Permutation method initial matrices illustration. Top: $A^{(0)}$ showing arrangement of edge entries (all solo connections: $\rho_m^+ = 0$) for each row aligned left where row sums add up to k_{in}. If multi-edges are desired, we simply distribute them in the rows of $A^{(0)}$ satisfying the proportion, $\rho_m^+ = 0$ and the row sum. Bottom: permutation of rows in $A^{(0)}$ into this example $A^{(1)}$. Note row sums still add up to k_{in}, with column sums adding to a current $k_{out}^{(1)}$ — likely violating the designated k_{out}.

[4] Fan Chung and Linyuan Lu. Connected components in random graphs with given expected degree sequences. *Annals of combinatorics*, 6(2):125–145, 2002.

[5] BC Coutinho, AV Goltsev, SN Dorogovtsev, and JFF Mendes. Kuramoto model with frequency-degree correlations on complex networks. *Physical Review E*, 87(3):032106, 2013.

[6] Daniel de Santos-Sierra, Irene Sendiña-Nadal, Inmaculada Leyva, Juan A Almendral, Sarit Anava, Amir Ayali, David Papo, and Stefano Boccaletti. Emergence of small-world anatomical networks in self-organizing clustered neuronal cultures. *PloS one*, 9(1):e85828, 2014.

[7] Victor M. Eguíluz, Dante R. Chialvo, Guillermo A. Cecchi, Marwan Baliki, and A. Vania Apkarian. Scale-free brain functional networks. *Phys. Rev. Lett.*, 94:018102, Jan 2005.

[8] Bard Ermentrout. Type I membranes, phase resetting curves, and synchrony. *Neural Computation*, 8(5):979–1001, 1996.

[9] G B Ermentrout and N Kopell. Parabolic bursting in an excitable system coupled with a slow oscillation. *SIAM Journal on Applied Mathematics*, 46(2):233–253, 1986.

[10] Jacob G. Foster, David V. Foster, Peter Grassberger, and Maya Paczuski. Edge direction and the structure of networks. *PNAS USA*, 107(24):10815–10820, 2010.

[11] Willy JF Govaerts. *Numerical methods for bifurcations of dynamical equilibria*, volume 66. Siam, 2000.

[12] Takashi Ichinomiya. Frequency synchronization in a random oscillator network. *Physical Review E*, 70(2):026116, 2004.

Algorithm 2: Permutation Method, Phase (1).
Pseudo-code for generating initial permutation matrix $A^{(0)}$ *satisfying* k_{in} *and* ρ_m^+ *yet violating* k_{out}.

```
/* compute number of multi-edges per node                                    */
k_m^+ = ρ_m^+ * k_in ;
for i = 1...N do
    /* assemble vector possible edge values                                  */
    e_i = [1, ..., max(k_m^+(i))] ;
    /* pick random edge values until sum of picks = k_in(i)                  */
    while sum(p) ≠ k_in do
        p_t = randpick(e_i) ;
        k_temp = sum([p p_t]) ;
        if k_temp ≤ k_in(i) then
            append p_t to p ;
        else
            reject p_t ;
        end
    end
    /* update A^(0)(i,:) with row vector p and zeros out to N                */
    A^(0)(i,:) = [p ... 0]_(1,N) ;
end
```


[13] M Kähne, IM Sokolov, and S Rüdiger. Population equations for degree-heterogenous neural networks. *Physical Review E*, 96(5):052306, 2017.

[14] C. R. Laing. Derivation of a neural field model from a network of theta neurons. *Physical Review E*, 90(1):010901, 2014.

[15] C. R. Laing. Numerical bifurcation theory for high-dimensional neural models. *The Journal of Mathematical Neuroscience*, 4(1):1, 2014.

[16] C. R. Laing. Exact neural fields incorporating gap junctions. *SIAM Journal on Applied Dynamical Systems*, 14(4):1899–1929, 2015.

[17] M Drew LaMar and Gregory D Smith. Effect of node-degree correlation on synchronization of identical pulse-coupled oscillators. *Physical Review E*, 81(4):046206, 2010.

[18] Peter E Latham, BJ Richmond, PG Nelson, and S Nirenberg. Intrinsic dynamics in neuronal networks. i. theory. *Journal of neurophysiology*, 83(2):808–827, 2000.

[19] Ashok Litwin-Kumar and Brent Doiron. Slow dynamics and high variability in balanced cortical networks with clustered connections. *Nature neuroscience*, 15(11):1498, 2012.

[20] Tanushree B Luke, Ernest Barreto, and Paul So. Complete classification of the macroscopic behavior of a heterogeneous network of theta neurons. *Neural Computation*, 25:3207–3234, 2013.

[21] Marijn B Martens, Arthur R Houweling, and Paul HE Tiesinga. Anti-correlations in the degree distribution increase stimulus detection performance in noisy spiking neural networks. *Journal of Computational Neuroscience*, 42(1):87–106, 2017.

[22] Warren S. McCulloch and Walter Pitts. The statistical organization of nervous activity. *Biometrics*, 4(2):91–99, 1948.

[23] Ernest Montbrió, Diego Pazó, and Alex Roxin. Macroscopic description for networks of spiking neurons. *Physical Review X*, 5(2):021028, 2015.

[24] M. E. J. Newman. Assortative mixing in networks. *Phys. Rev. Lett.*, 89:208701, Oct 2002.

Algorithm 3: Permutation Method, Phase (2).

Pseudo-code for permuting initial matrix $A^{(0)}$ into final matrix $A^{(n)}$ satisfying all constraints, k_{in}, ρ_m^+ and k_{out}.

```
/* permute entries of A^(0) into A^(1)                                   */
for i = 1...N do
    A^(1)(i,:) = randperm(A^(0)(i,:)) ;
end
/* compute deviation from target k_out                                   */
e_out^(1) = k_out^(1) - k_out ;
e_norm^(1) = norm(e_out^(1))_L2 ;
/* classify node deviation type                                          */
d^(1) = e_out^(1) > 0 ;
r^(1) = e_out^(1) < 0 ;
/* loop over donors until target k_out reached                           */
while e_norm^(i) ≠ 0 do
    for j over d^(i) do
        /* pick suitable pair donor and recipient entries                */
        while swap pair unsuitable do
            k = randpick(A^(i)(:,j)) ;
            /* if autoconnects disabled, remove this column from
               recipient list                                            */
            if no autoconnects then
                r^(i) = remove(r^(i), k) ;
            end
            l = randpick(r^(i)) ;
            if A^(i)(k,j) ≥ A^(i)(k,l) then
                swap pair suitable ;
            end
        end
        /* exchange edge from donor to recipient                         */
        A^(i)(k,j) = A^(i)(k,j) - 1 ;
        A^(i)(k,l) = A^(i)(k,l) + 1 ;
        /* update error tracking                                         */
        e_out^(i) = k_out^(i) - k_out ;
        e_norm^(i) = norm(e_out^(i))_L2 ;
        /* update recipient list                                         */
        r^(i) = e_out^(i) < 0 ;
    end
    /* update donor list                                                 */
    d^(i) = e_out^(i) > 0 ;
end
```

[25] M.E.J. Newman. The structure and function of complex networks. *SIAM Review*, 45(2):167–256, 2003.

[26] Duane Q. Nykamp, Daniel Friedman, Sammy Shaker, Maxwell Shinn, Michael Vella, Albert Compte, and Alex Roxin. Mean-field equations for neuronal networks with arbitrary degree distributions. *Phys. Rev. E*, 95:042323, Apr 2017.

[27] E. Ott and T.M. Antonsen. Low dimensional behavior of large systems of globally coupled oscillators. *Chaos*, 18:037113, 2008.

[28] Juan G Restrepo and Edward Ott. Mean-field theory of assortative networks of phase oscillators. *Europhysics Letters*, 107(6):60006, 2014.

[29] Alex Roxin. The role of degree distribution in shaping the dynamics in networks of sparsely connected spiking neurons. *Frontiers in Computational Neuroscience*, 5:8, 2011.

[30] Christian Schmeltzer, Alexandre Hiroaki Kihara, Igor Michailovitsch Sokolov, and Sten Rüdiger. Degree correlations optimize neuronal network sensitivity to sub-threshold stimuli. *PloS One*, 10:e0121794, 2015.

[31] Hesam Setareh, Moritz Deger, Carl CH Petersen, and Wulfram Gerstner. Cortical dynamics in presence of assemblies of densely connected weight-hub neurons. *Frontiers in computational neuroscience*, 11:52, 2017.

[32] Per Sebastian Skardal, Juan G Restrepo, and Edward Ott. Frequency assortativity can induce chaos in oscillator networks. *Physical Review E*, 91(6):060902, 2015.

[33] Per Sebastian Skardal, Jie Sun, Dane Taylor, and Juan G Restrepo. Effects of degree-frequency correlations on network synchronization: Universality and full phase-locking. *Europhysics Letters*, 101(2):20001, 2013.

[34] Bernard Sonnenschein, Francesc Sagués, and Lutz Schimansky-Geier. Networks of noisy oscillators with correlated degree and frequency dispersion. *The European Physical Journal B*, 86(1):12, 2013.

[35] JC Vasquez, AR Houweling, and P Tiesinga. Simultaneous stability and sensitivity in model cortical networks is achieved through anti-correlations between the in- and out-degree of connectivity. *Frontiers in Computational Neuroscience*, 7:156, 2013.

[36] Marina Vegué and Alex Roxin. Firing rate distributions in spiking networks with heterogeneous connectivity. *Phys. Rev. E*, 100:022208, Aug 2019.

E-mail address: c.blasche@massey.ac.nz

School of Natural and Computational Sciences, Massey University, Private Bag 102-904 NSMC, Auckland, New Zealand.

E-mail address: S.Means@massey.ac.nz

School of Natural and Computational Sciences, Massey University, Private Bag 102-904 NSMC, Auckland, New Zealand.

E-mail address: c.r.laing@massey.ac.nz

School of Natural and Computational Sciences, Massey University, Private Bag 102-904 NSMC, Auckland, New Zealand., phone: +64-9-414 0800 extn. 43512 fax: +64-9-4418136
